# Slow insertion of silicon probes improves the quality of acute neuronal recordings

**DOI:** 10.1038/s41598-018-36816-z

**Published:** 2019-01-14

**Authors:** Richárd Fiáth, Adrienn Lilla Márton, Ferenc Mátyás, Domonkos Pinke, Gergely Márton, Kinga Tóth, István Ulbert

**Affiliations:** 1 Institute of Cognitive Neuroscience and Psychology, Research Centre for Natural Sciences, Hungarian Academy of Sciences, Magyar tudósok körútja 2, H-1117 Budapest, Hungary; 2 Faculty of Information Technology and Bionics, Pázmány Péter Catholic University, Práter utca 50/A, H-1083 Budapest, Hungary; 3 Department of Anatomy and Histology, University of Veterinary Medicine, István utca 2, H-1078 Budapest, Hungary; 4 Institute of Experimental Medicine, Hungarian Academy of Sciences, Szigony utca 43, H-1083 Budapest, Hungary

## Abstract

Neural probes designed for extracellular recording of brain electrical activity are traditionally implanted with an insertion speed between 1 µm/s and 1 mm/s into the brain tissue. Although the physical effects of insertion speed on the tissue are well studied, there is a lack of research investigating how the quality of the acquired electrophysiological signal depends on the speed of probe insertion. In this study, we used four different insertion speeds (0.002 mm/s, 0.02 mm/s, 0.1 mm/s, 1 mm/s) to implant high-density silicon probes into deep layers of the somatosensory cortex of ketamine/xylazine anesthetized rats. After implantation, various qualitative and quantitative properties of the recorded cortical activity were compared across different speeds in an acute manner. Our results demonstrate that after the slowest insertion both the signal-to-noise ratio and the number of separable single units were significantly higher compared with those measured after inserting probes at faster speeds. Furthermore, the amplitude of recorded spikes as well as the quality of single unit clusters showed similar speed-dependent differences. Post hoc quantification of the neuronal density around the probe track showed a significantly higher number of NeuN-labelled cells after the slowest insertion compared with the fastest insertion. Our findings suggest that advancing rigid probes slowly (~1 µm/s) into the brain tissue might result in less tissue damage, and thus in neuronal recordings of improved quality compared with measurements obtained after inserting probes with higher speeds.

## Introduction

The application of electrophysiological recording techniques led to numerous major discoveries in the field of neuroscience. A large fraction of these discoveries has been achieved by investigating the firing patterns of multiple single neurons recorded extracellularly by neural probes^1^. State-of-the-art silicon-based probes now allow to record the activity of tens to hundreds of neurons simultaneously *in vivo*, in various animal models^2–4^. Although the main factor behind the ever-increasing single unit yield is the rapid growth of the number of recording sites realized on these probes, other factors might also significantly contribute to this increase. Among others, the surgical techniques and the implantation procedure used, or the size and geometry of the recording device are only a few examples which may influence the quality of recordings, and thus the final number of single units available for further examinations^5,6^.

One critical phase of acute *in vivo* experiments, which might have a significant impact on the recording quality, is the surgical insertion of the implant into the brain. Inserting a rigid neural probe into the brain tissue will damage neurons, glial cells and blood vessels along the insertion path, as well as compromise the blood-brain barrier^5–7^. Injuring or killing many neurons close to the recording probe will decrease the number of potential cells of which activity might be monitored later during the experiment. Therefore, to obtain high-quality neural recordings both in acute and chronic setting, it is of crucial importance to minimize the extent of immediate tissue damage caused by the mechanical insertion of the probe. The degree of this insertion-related tissue damage depends principally on the physical properties of the neural probe (e.g. dimensions of the probe, shape of the tip, roughness of the probe surface) and the conditions of the implantation (e.g. speed or angle of insertion). The impact of the probe’s physical attributes on the degree of tissue damage or on the long-term response of the brain tissue is well studied, as well as the effect of probe design on the penetration mechanics^5,8–14^. In contrast, the conditions for an optimal insertion which minimizes tissue trauma are less known.

One important factor which might affect the degree of damage done to the tissue during implantation is the speed at which the probe is inserted^15–19^. Scientists working with extracellular multielectrodes generally use insertion speeds in the range of 1 µm/s to 1 mm/s for implantation (a comprehensive list of electrophysiological studies reporting the insertion speed is provided in Supplementary Table S1). Despite the wide range of insertion speeds, there is still no consensus on whether slower or faster insertions should be favored to obtain high-quality neuronal recordings. Results of previous reports assessing the physical effects of the insertion speed on the brain tissue showed that slower insertions (125 µm/s) may result in a higher degree of vascular damage, while during faster insertions (2 mm/s) a lower mean effective strain was measured^15,16^. Although a slower insertion seems to do more damage, very slow insertion speeds (<10 µm/s) may be advantageous compared with faster speeds because these might provide the surrounding tissue time to accommodate around the probe and allow blood vessels enough time to recover without subsequent rupture^10,15^. However, despite the great interest in the impact of the insertion speed on the brain tissue, to the authors’ knowledge there are no reports which directly assess the quality of neuronal recordings obtained in an acute manner (1–2 hours) after implanting neural probes with various speeds.

In this study, we investigated the effect of the insertion speed on the quality of the recorded neuronal activity within 45 minutes of probe insertion. Four speeds were tested in the range of 2 µm/s to 1 mm/s. Spontaneously occurring neocortical activity of anesthetized rats was obtained using high-density, single-shank silicon-based probes with two different shaft thicknesses (15 and 50 µm). Quantitative assessment of the recorded spiking activity was performed by calculating the signal-to-noise ratio of recordings, as well as the single unit yield and various attributes of well-separated single units. Additionally, the neuronal cell loss around the probe track was quantified using image analysis on NeuN-immunostained brain sections. Our findings suggest that, within the range of the tested insertion speeds, very slow insertion of stiff silicon probes might result in a significantly better recording quality.

## Results

### Qualitative examination of the measured neuronal activity revealed insertion speed-dependent differences in the recording quality

**Figure 1:**
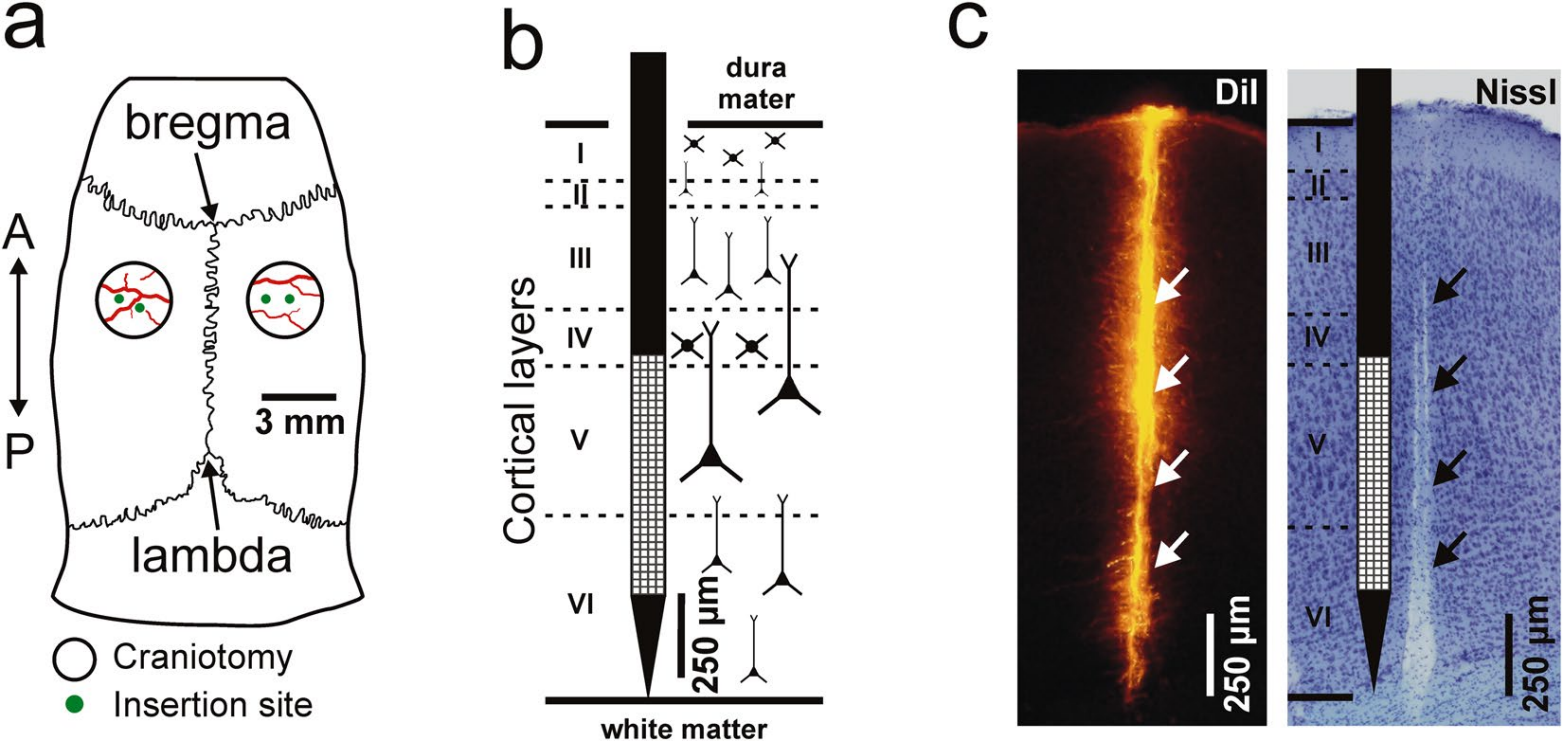
Schematic illustration of the experimental design. (**a**) The position of the four insertion sites (green dots) of a representative experiment shown on the schematic of a rat skull. The two craniotomies (hollow black circles) were prepared over the trunk region of the primary somatosensory cortex (A, anterior; P, posterior). (**b**) Schematic demonstration of the approximate position of the high-density silicon probe in the brain tissue after inserting it to a dorsoventral depth of 1700 µm. Small white squares mark the recording sites. Boundaries between cortical layers (roman numerals) are indicated with dashed lines. Note that most of the recording sites were located in layer V. (**c**) Representative coronal brain section prepared from the right brain hemisphere. On the left, fluorescent marks of DiI are visible indicating the track of the probe. On the right, the same brain section after Nissl-staining. Arrows indicate the position of the probe track, while dashed lines mark cortical layer boundaries.

To examine whether the speed used to insert rigid neural probes into the brain tissue might influence the quality of brain electrical activity recorded *in vivo*, we acutely implanted high-density silicon-based probes at four different insertion speeds (0.002 mm/s, 0.02 mm/s, 0.1 mm/s, 1 mm/s) into four separate locations of the somatosensory cortex of ten rats anesthetized with ketamine/xylazine (Fig. 1a). The silicon probe used in the study had a single 50 µm thick and 100 µm wide shank which contained 128 closely-packed recording sites^2^. The square-shaped (20 × 20 µm^2^) recording sites with 2.5 µm spacing between them formed a 32 × 4 dense array (Fig. 1b). In case the dura mater is removed properly above the insertion site, the chisel-shaped tip of this specific probe provides a smooth penetration into the brain tissue with minimal brain dimpling and minor tissue damage^2^.

To exclude any contribution of the order of the insertion speeds on the quality of recordings, the sequence of the four speeds was randomized between experiments. After each penetration, we recorded the wideband (0.1–7500 Hz) spontaneous cortical activity for 45 minutes, starting immediately after the tip of the probe reached the specified implantation depth (1700 µm from the top of the cortex).

The wideband extracellular neural activity recorded with invasive probes is usually separated into local field potentials and action potentials of individual neurons (i.e. multi-unit and single-unit activities). Local field potentials measured in the lower (<500 Hz) frequency band are generated by the combined activity of large populations of neurons. Among these neurons, a substantial number of cells are located several hundreds of micrometers away from the implanted probe^20^. Hence, the quality of the acquired local field potentials is relatively robust to the acute tissue trauma caused by the probe insertion. In contrast, action potentials (or also called spikes) detected by the recording device are fired by individual neurons which are located close (<150 µm) to the implanted probe^1^. Thus, these cells are more exposed to the insertion-related tissue damage. Therefore, to reliably detect differences in the quality of recordings related to the insertion speed or alterations in the recording quality over time, we limited the scope of this work to the examination of the frequency band containing the spikes of neurons. To extract spikes from the wideband recordings, unwanted frequency components were removed using a band-pass filter with cutoff frequencies of 500 and 5000 Hz.

**Figure 2:**
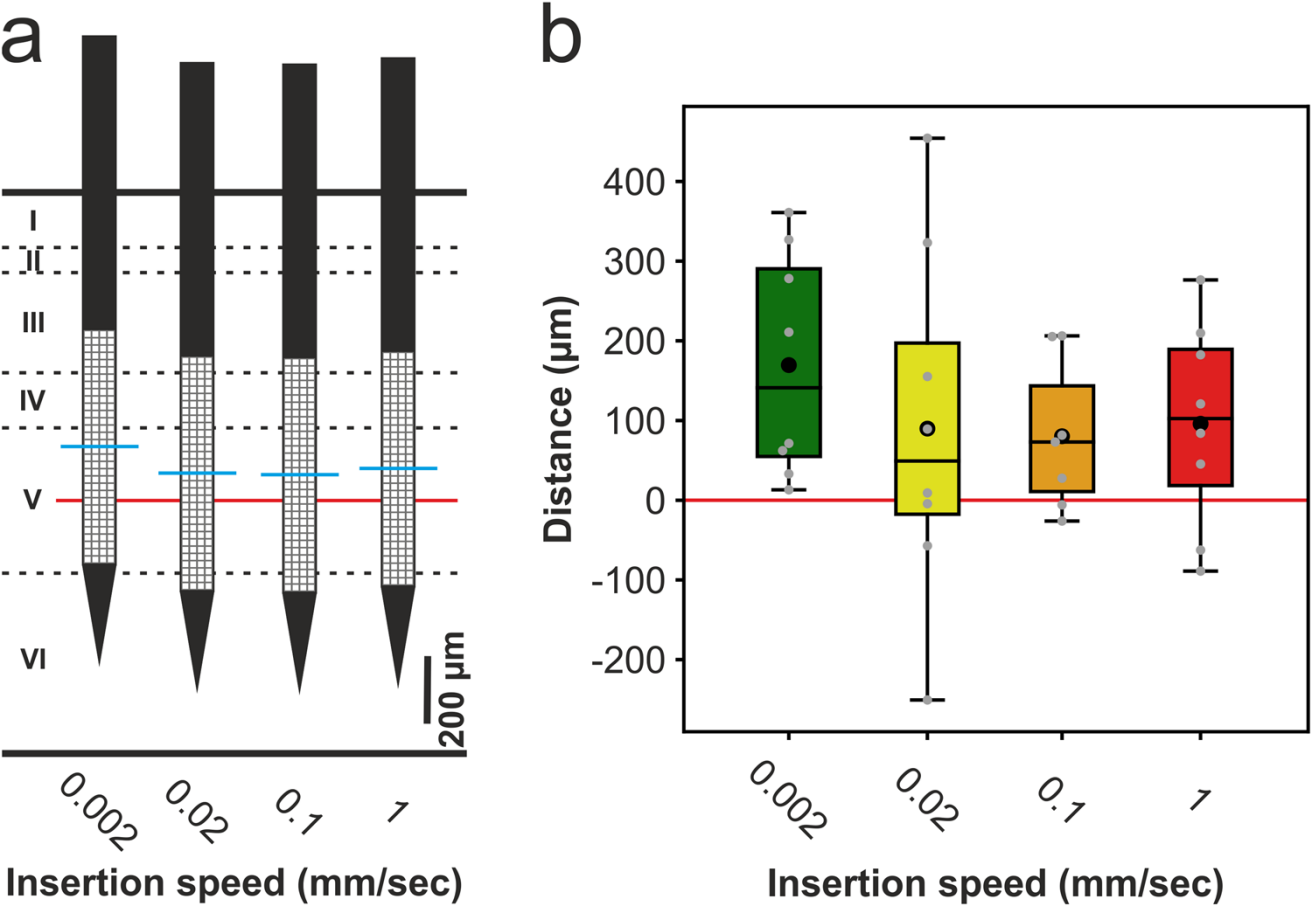
Estimated position of the recording sites relative to cortical layer V for each insertion speed. (**a**) The average distance between the depth corresponding to the middle of layer V (red line) and the depth corresponding to the middle of the electrode array (blue line) for each insertion speed. The depth of the insertion as well as the location of layer V was estimated by examining the coronal brain sections. Cortical layers are indicated with roman numerals. (**b**) Box-and-whisker plot of the distances between the middle of layer V (red line) and the middle of the electrode array for each insertion speed. On the box-and-whisker plot, the middle line indicates the median, while the boxes correspond to the 25th and 75th percentile. Whiskers mark the minimum and maximum values. The average is depicted with a black dot. Gray dots correspond to distance values obtained for individual penetrations (8 penetrations for speeds: 0.002 mm/s, 0.02 mm/s and 1 mm/s; 7 penetrations for speed: 0.1 mm/s). The distance values were not significant between insertion speeds (one-way ANOVA; p = 0.656).

With the microelectrodes of the used silicon probes we can cover multiple (2–3) cortical layers but not all of them^2^. At the targeted cortical depth, the neural probe should record the electrical activity of granular and infragranular cortical layers (layer IV-VI; Fig. 1b,c), but mainly that of layer V. We chose this depth because, out of the six cortical layers, neurons located in layer V show the strongest and most robust action potential firing during the slow-wave activity induced by the anesthetic cocktail ketamine/xylazine^21^. In contrast, neuronal activity in supragranular layers (layer I-III) is sparse during slow-wave activity^21,22^. Thus, implanting the probes into the deep layers of the somatosensory cortex displaying higher levels of spontaneous activity allowed us a more reliable comparison of the recording quality between insertion speeds. To exclude the effect of the variation in the insertion depth on the results, we examined the relative position of cortical layer V and the recording sites of the probe. We found no systematic variation in insertion depth with the insertion speed and most of the sites were located in layer V (one-way ANOVA, p = 0.656; Fig. 2).

**Figure 3:**
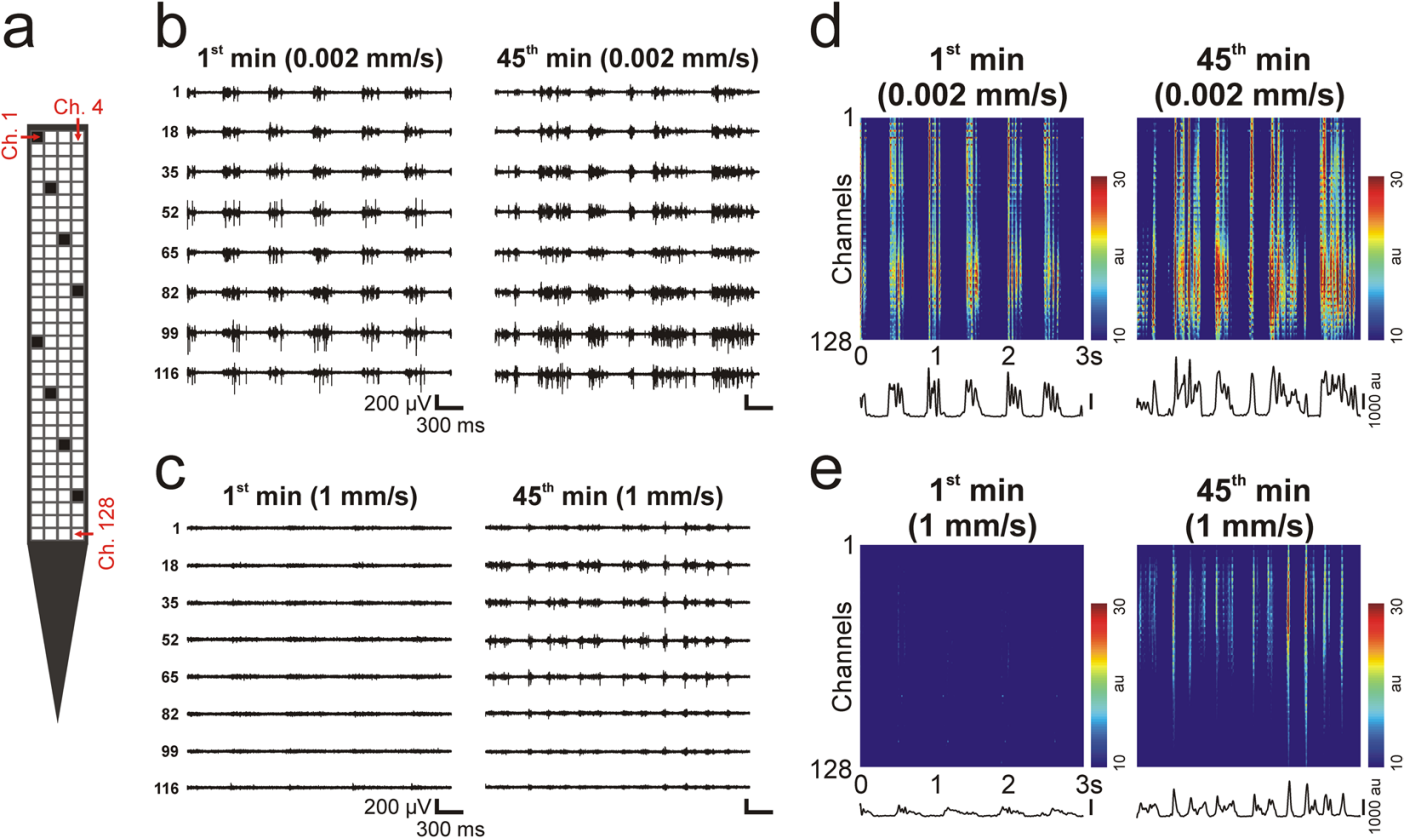
Quality of neuronal recordings after the slowest (0.002 mm/s) and the fastest (1 mm/s) probe insertion. (**a**) Schematic of the tip section of the silicon probe comprising 128 close-packed electrodes (white and black squares), with the electrode–channel relationship indicated (red text; Ch, channel). Representative three-second-long multi-unit activity (MUA) traces recorded after slow (**b**) and after fast (**c**) insertion on eight electrodes (colored black in panel (a)). Traces on the left show MUA shortly after probe insertion (1^st^ min), while traces on the right were obtained 45 minutes after implantation (45^th^ min). The same voltage scale was used on all the traces. Color maps constructed from three-second-long data recorded on all channels after slow (**d**) and after fast (**e**) insertion. Color maps on the left show MUA shortly after probe insertion (1^st^ min), while color maps on the right were constructed from data recorded 45 minutes after implantation (45^th^ min). Before plotting the color maps, the absolute value of the MUA was calculated, then a 30 Hz low-pass filter was applied on the data to obtain the envelope of the MUA (au; arbitrary unit). Black traces below the color maps show the instantaneous population activity recorded by the probe at each sample point of the three seconds, which was obtained by summing the values on all of the channels.

Visual inspection of the quality of the acquired data showed the most remarkable differences between neuronal activity recorded after the slowest (0.002 mm/s) and the fastest (1 mm/s) probe insertion (Fig. 3). In most of the cases, the multi-unit activity recorded within the first minute after the slowest insertion (Fig. 3b,d) was noticeably stronger compared with that obtained after inserting the neural probe with the fastest speed (Fig. 3c,e). Furthermore, the two alternating states characteristic of the slow-wave activity (i.e. active states with high spiking activity and inactive states with temporarily ceased action potential firing) could be clearly distinguished by visual examination on most of the recording channels after the slow insertion, while these were hard to identify in the data recorded after fast insertion (Fig. 3b–e). Usually, the quality of recordings obtained after the slow insertion changed only slightly during the investigated 45 minutes (Fig. 3b,d). In contrast, the quality of multi-unit activity observed after fast insertion improved remarkably during the recording period (Fig. 3c,e). In general, however, the quality of measurements corresponding to the slowest and fastest insertion speed was still different at the end of the recordings, with measurements obtained after the slowest insertion showing higher levels of population activity. The quality of recordings obtained after probe implantation with the two intermediate insertion speeds (0.02 mm/s and 0.1 mm/s) was usually between the quality of the data demonstrated in Fig. 3.

### Inserting the silicon probe with the slowest speed provided neuronal signals with the highest and most stable signal-to-noise ratio

To quantitatively assess the quality of the recorded neuronal data, we calculated the signal-to-noise ratio (SNR) of recordings. The SNR is a measure suitable to determine the quality of neuronal signals or to track the recording capabilities of a neural implant over time^23–25^. For each penetration, the SNR for each 30-second-long segment of data was determined on each recording channel, then the computed values were averaged across channels. Finally, the SNR was compared across insertion speeds (number of computed SNR values after data cleaning for each speed: 0.002 mm/s, n = 897; 0.02 mm/s, n = 867; 0.1 mm/s, n = 802; 1 mm/s, n = 753).

**Figure 4:**
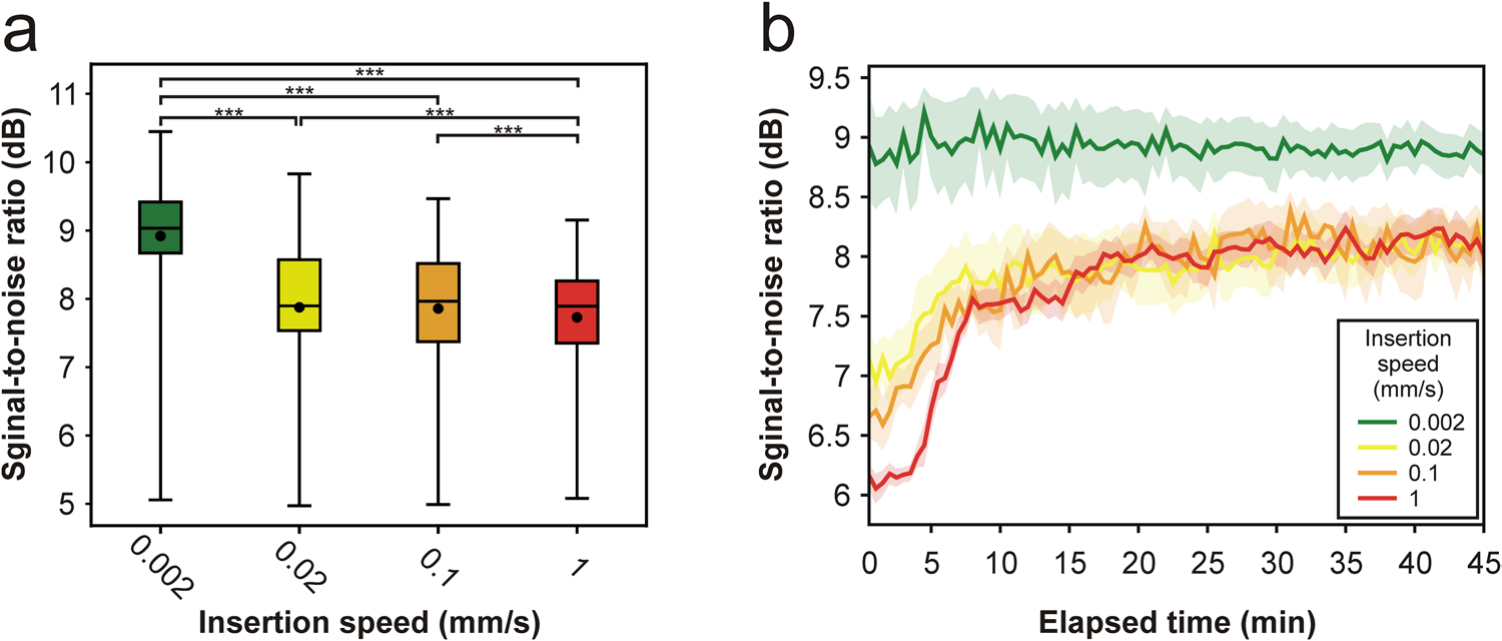
Neuronal activity obtained after probe insertion with the slowest speeds showed the highest and most stable signal-to-noise ratio. (**a**) Box-and-whisker plot of the signal-to-noise ratio (SNR) values for each insertion speed. SNR values were calculated from consecutive, 30-second-long segments of the recordings, during the entire 45-minute-long recording period, then averaged across channels (number of computed SNR values after data cleansing for each speed: 0.002 mm/s, n = 897; 0.02 mm/s, n = 867; 0.1 mm/s, n = 802; 1 mm/s, n = 753). On the box-and-whisker plot, the middle line indicates the median, while the boxes correspond to the 25th and 75th percentile. Whiskers mark the minimum and maximum values. The average is depicted with a black dot. ***p < 0.001; Dunn’s post-hoc test with Bonferroni correction. (**b**) Change in the average SNR of the recorded spiking activity over time for each insertion speed. Colored bands correspond to the standard error of mean.

We found significant differences in the SNR among different insertion speeds (Kruskal-Wallis test, p < 1 × 10^−18^). Pairwise statistical comparisons showed that the SNR was significantly higher after the slowest probe implantation compared with those computed for faster speeds (Fig. 4a; 0.002 mm/s vs. 0.02 mm/s, p < 1 × 10^−18^; 0.002 mm/s vs. 0.1 mm/s, p < 1 × 10^−18^; 0.002 mm/s vs. 1 mm/s, p < 1 × 10^−18^). Similarly, the SNR values calculated for the two intermediate speeds (0.02 mm/s and 0.1 mm/s) were significantly higher compared with those measured after the insertions at 1 mm/s (Fig. 4a; 0.02 mm/s vs. 1 mm/s, p = 9 × 10^−5^; 0.1 mm/s vs. 1 mm/s, p = 9.72 × 10^−4^).

To assess how the recording quality changes over time and whether there are differences in the time course of the recording quality between the four insertion speeds, we examined temporal alterations in the SNR (Fig. 4b). The time course of the average SNR was remarkably different between the slowest (2 µm/s) and the other three, faster insertion speeds. After the slowest insertion, a relatively high SNR level (~ 9 dB) was measured at the beginning of the recordings (Fig. 4b). During the 45 minutes, the SNR remained at around the same level until the end of data acquisition. In contrast, the SNR measured after the three faster insertions started at lower levels (6–7 dB), with higher starting SNR levels corresponding to slower insertions (Fig. 4b). Then, during the first 15 minutes of recordings, the SNR showed a marked increase, reaching about the same level (~8 dB) for each of the three faster speeds. After that, the SNR stayed at around this level until the end of the recording period. Although we investigated neuronal signals only within the first 45 minutes after insertion, based on the SNR, the recordings are relatively stable also over longer periods of time (3–4 hours; Supplementary Fig. S1).

### The insertion speed significantly affected the single unit yield and the quality of separated neuron clusters

The investigation of firing patterns of individual neurons (or single units) is a fundamental method in the field of neuroscience. Therefore, a high single unit yield (i.e. the number of individual neurons which can be extracted from a single experiment) is of crucial importance for neuroscientists.

**Figure 5:**
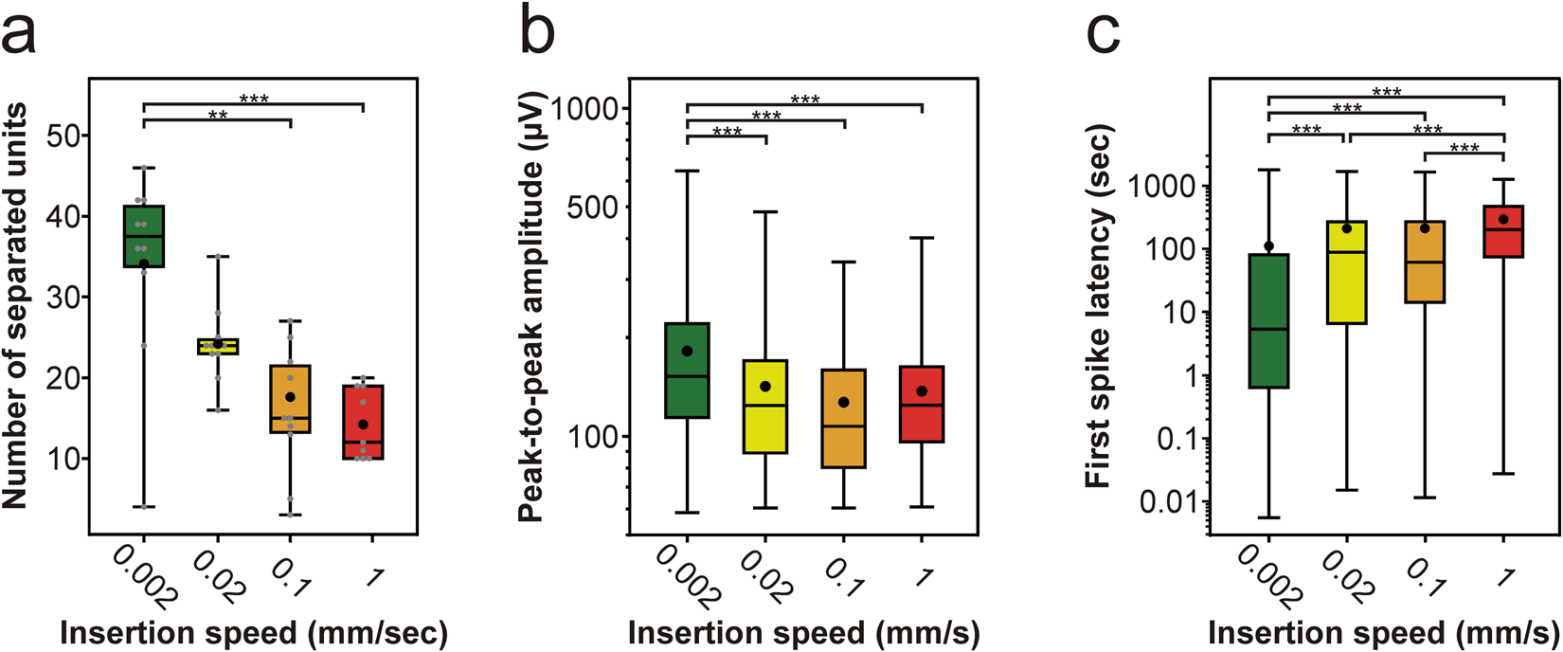
Insertion speed-dependent difference in the properties of the recorded single-unit activity. (**a**–**c**) Box-and-whisker plots showing the distribution of the number of well-separated single unit clusters (**a**), the distribution of the peak-to-peak amplitude of spike waveforms (**b**), and the distribution of the first spike latencies (**c**) for each insertion speed (total number of well-separated neurons for each speed: 0.002 mm/s, n = 341; 0.02 mm/s, n = 242; 0.1 mm/s, n = 159; 1 mm/s, n = 128). On the box-and-whisker plots, the middle line indicates the median, while the boxes correspond to the 25th and 75th percentile. Whiskers mark the minimum and maximum values. The average is depicted with a black dot. Gray dots on panel (a) correspond to single unit yields obtained for individual penetrations. Data on panel (b) and (c) are plotted on a logarithmic scale. **p < 0.01; ***p < 0.001; Dunn’s post-hoc test with Bonferroni correction.

To assess the relationship between the single unit yield and the insertion speed, we performed spike sorting on the recorded data. Only neuron clusters with a clear refractory period of 2 ms and with an averaged spike waveform having a peak-to-peak amplitude over 60 µV were used for further analysis. Our results show that the average number of well-separated single units per penetration negatively correlated with the insertion speed (Fig. 5a; Table 1). The single unit yield was the highest when we inserted the probe with the slowest speed (2 µm/s), it was lower at faster speeds, reaching the lowest unit number when the fastest (1 mm/s) speed was applied for insertion. The difference in the single unit yield was statistically significant between insertion speeds (Kruskal-Wallis test, p = 2.14 × 10^−4^). Post-hoc tests revealed that the number of single units was significantly higher after the slowest insertion compared with those obtained after the two fastest insertions (Fig. 5a; Table 1; 0.002 mm/s vs. 0.1 mm/s, p = 9.05 × 10^−3^; 0.002 mm/s vs. 1 mm/s, p = 8.13 × 10^−4^). Furthermore, the peak-to-peak amplitude of the average spike waveform of these well-separated neurons was significantly different across insertion speeds (Kruskal-Wallis test, p = 1.04 × 10^−14^). Pairwise comparisons showed that the spike amplitudes of single units recorded after the slowest insertion were significantly higher compared with those measured after faster insertions (Fig. 5b; Table 1; 0.002 mm/s vs. 0.02 mm/s, p = 4.33 × 10^−8^; 0.002 mm/s vs. 0.1 mm/s, p = 1.85 × 10^−12^; 0.002 mm/s vs. 1 mm/s, p = 1.2 × 10^−5^). Moreover, we found a significant difference in the latency of the first spike fired by the investigated neurons (Kruskal-Wallis test, p < 1 × 10^−18^). Pairwise comparisons revealed that single units extracted with spike sorting after the slowest insertion started to fire significantly earlier compared with those units which were separated from data obtained after faster insertions (Fig. 5c; Table 1; 0.002 mm/s vs. 0.02 mm/s, p = 1.41 × 10^−11^; 0.002 mm/s vs. 0.1 mm/s, p = 1.83 × 10^−10^; 0.002 mm/s vs. 1 mm/s, p < 1 × 10^−18^). Likewise, the first spike latencies calculated for the two intermediate speeds were significantly shorter compared with those computed for the fastest insertion (Fig. 5c; Table 1; 0.02 mm/s vs. 1 mm/s, p = 1.4 × 10^−5^; 0.1 mm/s vs. 1 mm/s, p = 4.84 × 10^−4^). In summary, the above findings suggest that, compared to faster insertions, slower probe insertions might provide recordings with a higher number of single units, which units have larger spike amplitudes and start to fire earlier, shortly after the probe reached the insertion depth.

**Table 1:** Calculated properties of well-isolated single units obtained with the 128-channel silicon probe grouped according to insertion speed (average ± standard deviation).

Properties	0.002 mm/s	0.02 mm/s	0.1 mm/s	1 mm/s
Total number of separated single units	341	242	159	128
Number of separated single units per penetration	34.1 ± 12.2	24.2 ± 4.9	15.9 ± 7.9	14.2 ± 4.4
Peak-to-peak amplitude (µV)	182.1 ± 99.4	142.1 ± 71.6	127.1 ± 59.6	137.3 ± 63.0
First spike latency (s)	110.9 ± 246.0	209.5 ± 325.5	210.8 ± 329.9	294.4 ± 284.4
Isolation distance	43.8 ± 40.8	40.6 ± 39.8	31.6 ± 27.4	26.6 ± 28.0
Violation rate (%)	0.14 ± 0.24	0.12 ± 0.20	0.10 ± 0.16	0.12 ± 0.18

**Figure 6:**
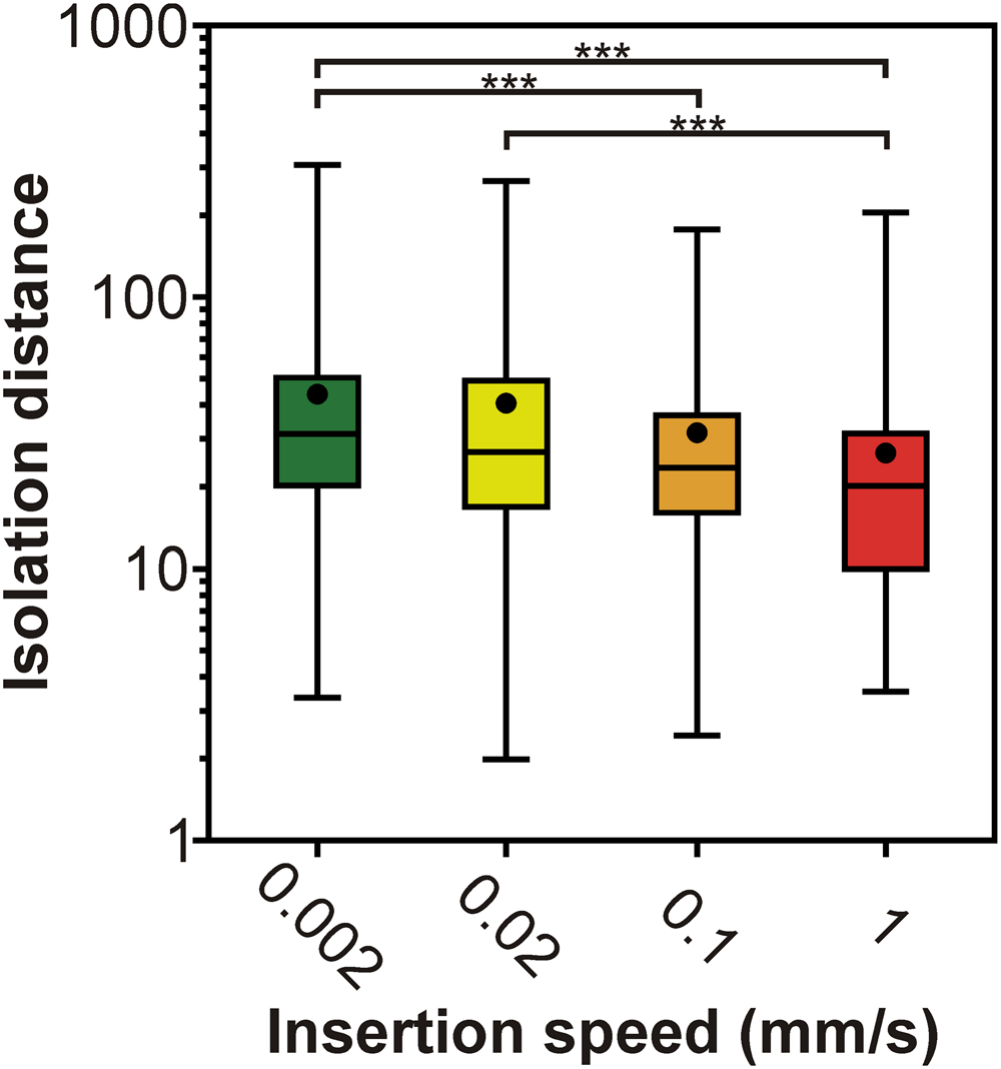
Insertion speed-dependent difference in the quality of single unit clusters. Box-and-whisker plots showing the distribution of the isolation distance of well-separated single unit clusters for each insertion speed (total number of well-separated neurons for each speed: 0.002 mm/s, n = 341; 0.02 mm/s, n = 242; 0.1 mm/s, n = 159; 1 mm/s, n = 128). On the box-and-whisker plots, the middle line indicates the median, while the boxes correspond to the 25th and 75th percentile. Whiskers mark the minimum and maximum values. The average is depicted with a black dot. Data are plotted on a logarithmic scale. ***p < 0.001; Dunn’s post-hoc test with Bonferroni correction.

Various quantitative cluster quality metrics are available to determine the quality of single unit clusters, like the isolation distance or the L-ratio^26,27^. For instance, the isolation distance indicates how well the spikes belonging to one neuron cluster are separated from other clusters^26^. To examine whether the insertion speed has an influence on the quality of the separated clusters, the isolation distance was calculated for each separated single unit (Fig. 6). We found a significant difference in the cluster quality between insertion speeds (Kruskal-Wallis test, p = 1.2 × 10^−10^). The results of post-hoc tests showed that the isolation distance of neuron clusters corresponding to the slowest insertion was significantly higher compared with those extracted after the two fastest insertions (Fig. 6; Table 1; 0.002 mm/s vs. 0.1 mm/s, p = 2.62 × 10^−4^; 0.002 mm/s vs. 1 mm/s, p = 2 × 10^−10^). Furthermore, the quality of neuron clusters obtained after inserting the probe with a speed of 0.02 mm/s was significantly better than that of neurons sorted after the fastest insertion (Fig. 6; Table 1; 0.02 mm/s vs. 1 mm/s, p = 5.2 × 10^−5^).

In addition to the isolation distance, for each single unit, we calculated the ratio of spikes violating the absolute refractory period of neurons (termed “violation rate”). The absolute refractory period is a time interval (~2 ms) during which the neuron cannot generate action potentials after it fired a spike. Therefore, a high violation rate (>5%), which is a sign of low cluster quality, indicates that spikes fired by other neurons contaminate the isolated neuron cluster. Since only well-isolated single units with a clear refractory period (violation rate < 2%) were used for analysis in the study, we expected that the violation rate will not depend on the speed of insertion. As we anticipated, no statistically significant difference was found in the violation rate between insertion speeds (Table 1; Kruskal-Wallis test, p = 0.294).

### The ratio of putative inhibitory interneurons was the highest after the slowest insertion

**Figure 7:**
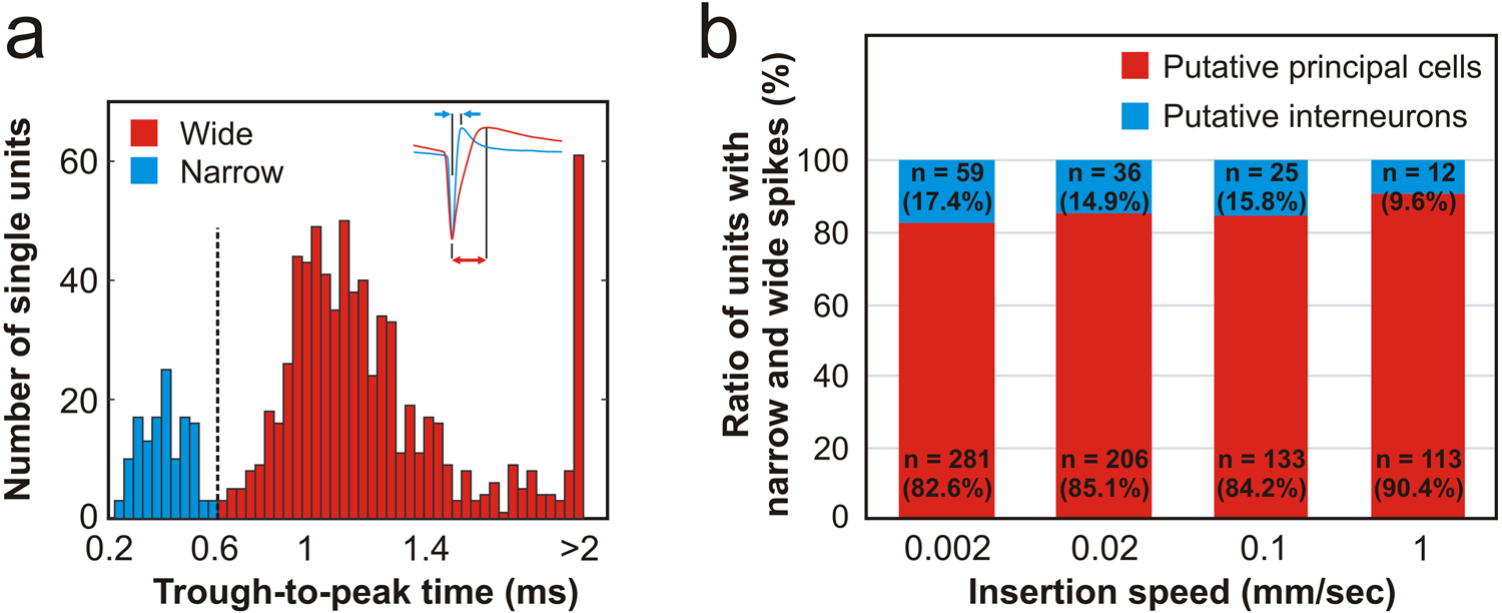
Ratio of putative interneurons and principal cells among insertion speeds. (**a**) Bimodal distribution of the trough-to-peak time of single unit spike waveforms. A threshold of 0.6 ms (vertical dashed line) was used to classify units either as narrow spiking (blue) or as wide spiking (red) neurons (i.e. as putative inhibitory interneurons or excitatory principal cells, respectively). A sample average spike waveform of each neuron type is shown in the inset. (**b**) Ratio of putative principal cells (wide spikes, red) and putative interneurons (narrow spikes, blue) calculated for each insertion speed. The number of neurons in each group is indicated.

Based on the duration of the extracellular spike waveforms, we can distinguish between putative excitatory neurons (principal cells) and inhibitory interneurons^28^. These two types of neurons have different roles in neural computations, therefore it is essential to identify and separate them. Spikes with a short duration (narrow spikes) are usually fired by GABAergic interneurons, while wide spikes are generated by principal cells (mostly by pyramidal cells). Based on the trough-to-peak time of their spike waveforms, the separated single units were classified as putative interneurons (n = 132) or principal cells (n = 733; Fig. 7a). Then, we assessed whether there are insertion speed-dependent differences in the ratio of these two cell types. According to our findings, the proportion of interneurons negatively correlated with the insertion speed (Fig. 7b). From single units obtained after the slowest insertions, more than 17% fired narrow spikes, while the ratio of putative interneurons was only 9.6% after the fastest insertion (Fig. 7b). The increase of the interneuron proportion toward slower speeds was tendentious but no statistical difference was found in the ratio of interneurons and principal cells between insertion speeds (two-tailed chi-squared test, p = 0.229). However, the above observations suggest that using slow insertion speeds in the range of 1 µm/s, besides providing recordings with more single units, may also be beneficial to record the activity of a higher proportion of inhibitory interneurons.

### Quality of neuronal recordings obtained using silicon probes with a thinner shaft

**Figure 8:**
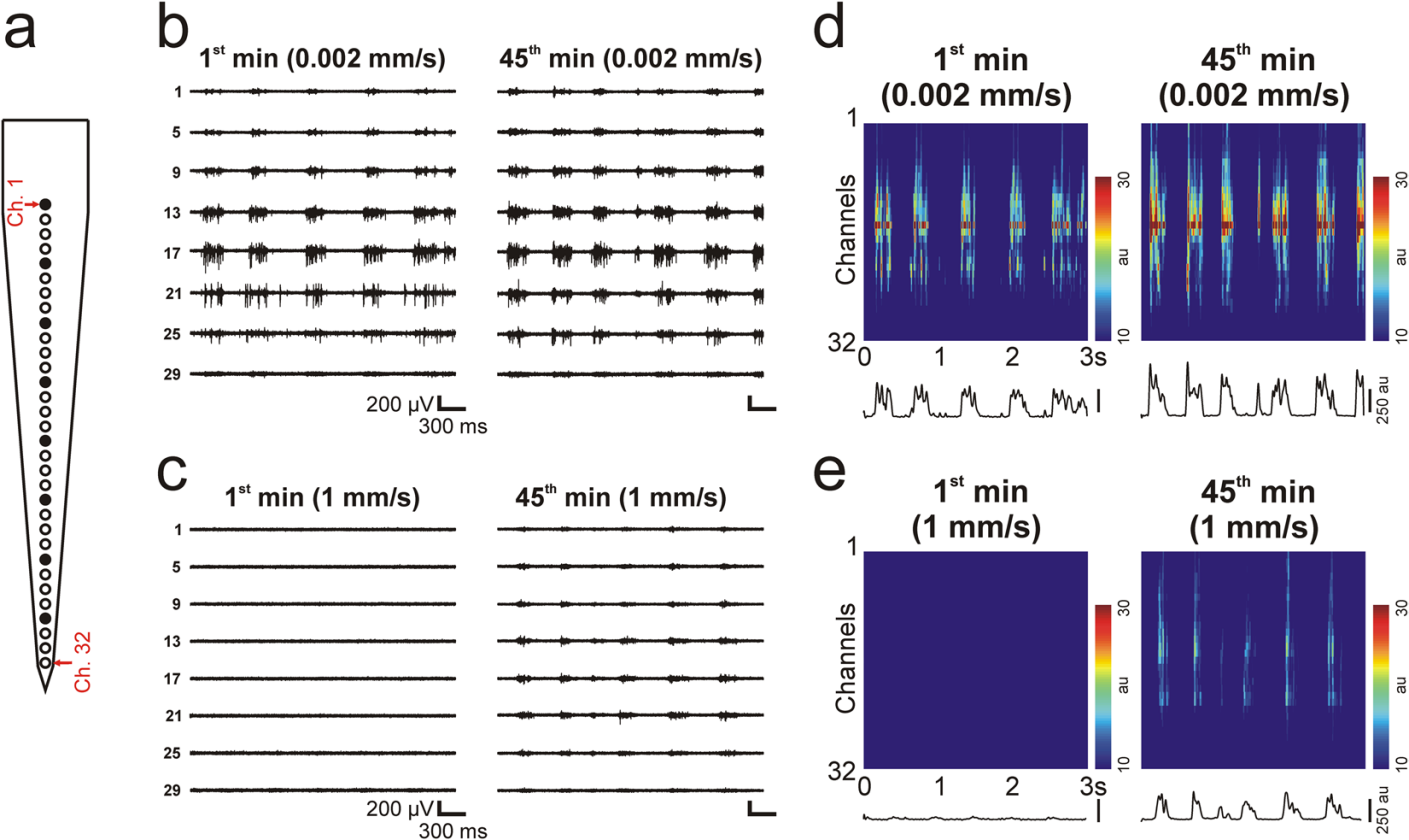
Quality of neuronal recordings after inserting the 15-µm-thick, 32-channel silicon probe with the slowest (0.002 mm/s) and the fastest (1 mm/s) speed. (**a**) Schematic of the tip section of the silicon probe comprising 32 electrodes (white and black squares), with the electrode–channel relationship indicated (red text; Ch, channel). Representative three-second-long multi-unit activity (MUA) traces recorded after slow (**b**) and after fast (**c**) insertion on eight electrodes (colored black in panel (a)). Traces on the left show MUA shortly after probe insertion (1^st^ min), while traces on the right were obtained 45 minutes after implantation (45^th^ min). The same voltage scale was used on all the traces. Color maps constructed from three-second-long data recorded on all channels after slow (**d**) and after fast (**e**) insertion. Color maps on the left show MUA shortly after probe insertion (1^st^ min), while color maps on the right were constructed from data recorded 45 minutes after implantation (45^th^ min). Before plotting the color maps, the absolute value of the MUA was calculated, then a 30 Hz low-pass filter was applied on the data to obtain the envelope of the MUA (au; arbitrary unit). Black traces below the color maps show the instantaneous population activity recorded by the probe at each sample point of the three seconds, which was obtained by summing the values on all of the channels.

**Figure 9:**
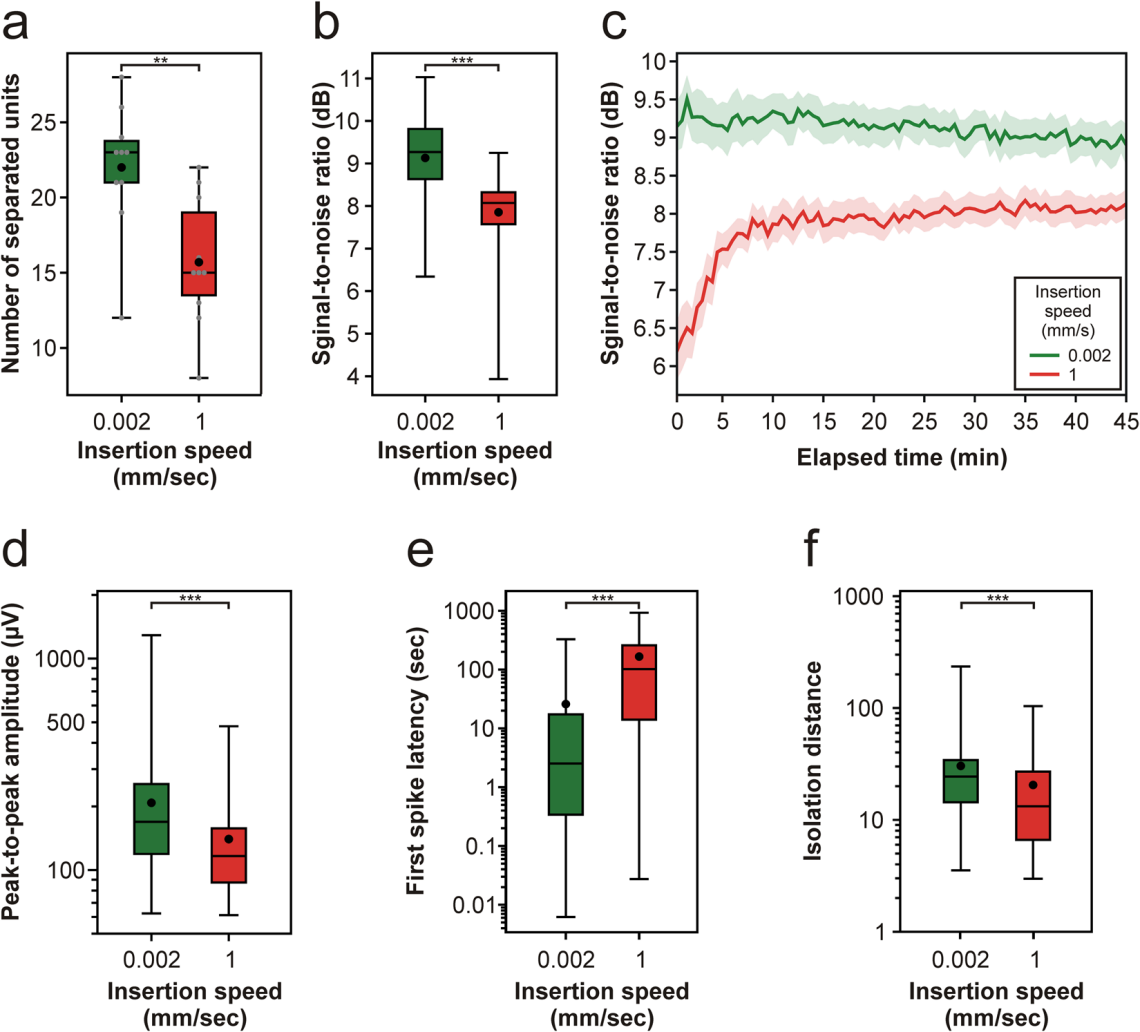
Insertion speed-dependent difference in the properties of the single-unit activity recorded with the 32-channel silicon probe. (**a**) Box-and-whisker plot showing the distribution of the number of well-separated single unit clusters. (**b**) Box-and-whisker plot of the signal-to-noise ratio (SNR) values for each insertion speed. SNR values were calculated from consecutive, 30-second-long segments of the recordings, during the entire 45-minute-long recording period, then averaged across channels (number of computed SNR values after data cleansing for each speed: 0.002 mm/s, n = 899; 1 mm/s, n = 896). (**c**) Change in the average SNR of the recorded spiking activity over time for each insertion speed. Colored bands correspond to the standard error of mean. (**d**–**f**) Box-and-whisker plot showing the distribution of the peak-to-peak amplitude of spike waveforms (**d**), the distribution of the first spike latencies (**e**), and the distribution of the isolation distances (**f**) for each insertion speed (total number of well-separated neurons for each speed: 0.002 mm/s, n = 220; 1 mm/s, n = 157). On the box-and-whisker plots, the middle line indicates the median, while the boxes correspond to the 25th and 75th percentile. Whiskers mark the minimum and maximum values. The average is depicted with a black dot. Gray dots on panel (a) correspond to single unit yields obtained for individual penetrations. Data on panels (d-f) are plotted on a logarithmic scale. **p < 0.01; ***p < 0.001; Mann-Whitney U test.

To examine whether our results may be generalized to other probes types, we investigated the quality of recordings obtained with a different single-shank probe, namely a 32-channel linear silicon probe having a shaft thickness of only 15 µm and a recording site spacing of 25 µm. Since the results obtained with the thicker, 128-channel probe were relatively similar for the three faster insertions speeds, only the slowest (0.002 mm/s) and the fastest (1 mm/s) speeds were compared. Similar to the findings obtained with the thicker probe, there was a considerable difference in the quality of the recorded neuronal signals (Fig. 8). We found a significant difference between the two insertion speeds in the single unit yield (Fig. 9a; Table 2; Mann-Whitney U test; p = 3.89 × 10^−3^), in the SNR (Fig. 9b,c; Table 2; Mann-Whitney U test; p < 1 × 10^−18^), as well as in the peak-to-peak amplitude (Fig. 9d; Table 2; Mann-Whitney U test; p = 1.8 × 10^−9^), first spike latency (Fig. 9e; Table 2; Mann-Whitney U test; p < 1 × 10^−18^) and isolation distance (Fig. 9f; Table 2; Mann-Whitney U test; p = 2.4 × 10^−7^) of single units. Furthermore, the time course of the SNR corresponding to the two speeds was analogous to that observed with the thicker probe (Figs 4b vs. 9c). Finally, in the interneuron ratio, there was no difference between the two insertion speeds (0.002 mm/s: 34/220 units, 15.45%; 1 mm/s: 24/133 units, 15.29%). Thus, although the difference in the single unit yield between the insertion speeds was not as large as observed with the thicker probe, our findings suggest that using a slow insertion speed might be beneficial also in the case of less invasive probes having a smaller shaft cross-section.

**Table 2:** Calculated properties of well-isolated single units obtained with the 32-channel silicon probe grouped according to insertion speed (average ± standard deviation).

Properties	0.002 mm/s	1 mm/s
Total number of separated single units	220	157
Number of separated single units per experiment	22.0 ± 4.3	15.7 ± 4.3
Peak-to-peak amplitude (µV)	207.9 ± 145.9	140.1 ± 77.6
First spike latency (s)	25.9 ± 53.6	166.1 ± 196.7
Isolation distance	30.4 ± 29.4	20.5 ± 19.5
Violation rate (%)	0.18 ± 0.27	0.16 ± 0.27

### A reduced loss of neurons was observed around the probe track after slow insertion compared with fast insertion

**Figure 10:**
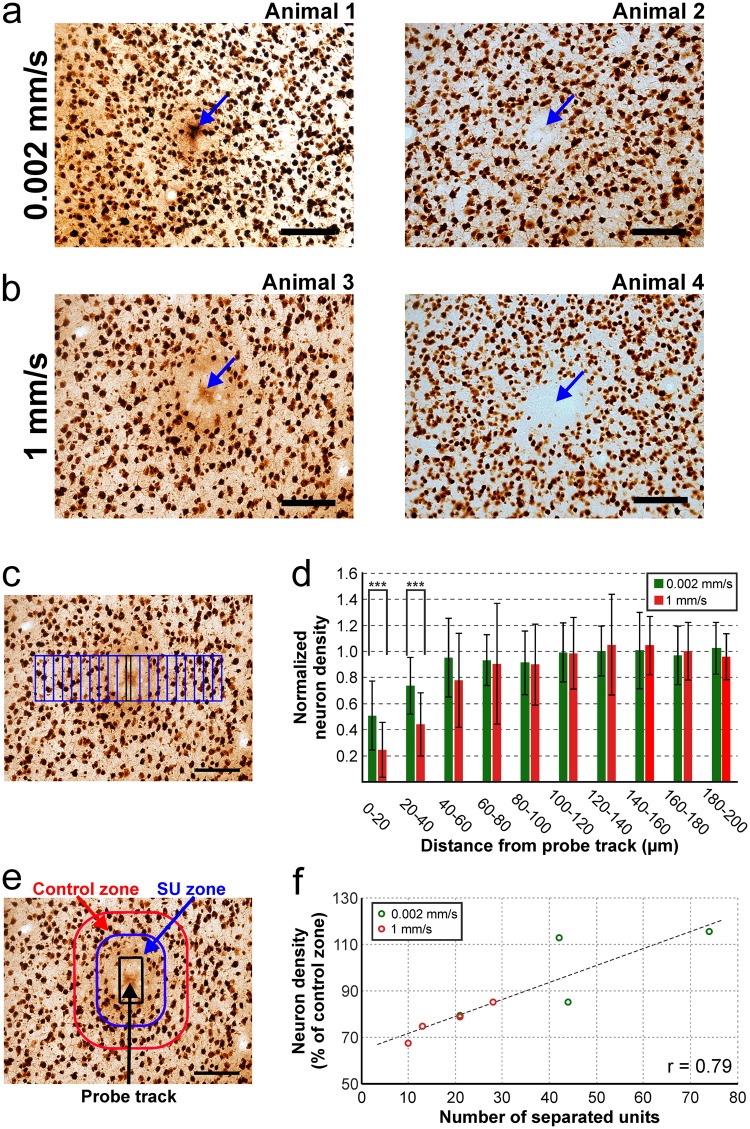
Insertion speed-dependent difference in the neuronal cell loss around the probe track. (**a**,**b**) Representative NeuN-stained horizontal brain sections showing the probe track (blue arrow) and nearby neurons (small dark patches) after penetrations done either with the slowest (**a**) or with the fastest (**b**) insertion speed. Brain sections from two animals are shown for each speed. 20-fold magnification. Scale bars = 100 µm. (**c**) Neuron numbers were automatically counted in twenty bins (blue rectangles having an area of 20 × 100 µm^2^) on each side of the probe track (black rectangle in the center; area: 10 × 100 µm^2^). Only ten bins are shown on each side. Scale bar = 100 µm. (**d**) Normalized neuron density in the first ten 20-µm-wide bin located closest to the probe track for each insertion speed after automatic image analysis. Average and standard deviation is presented. Normalized neuron densities were averaged across penetrations and sides. ***p < 0.001; Student’s t-test. (**e**) To complement the results of the automatic cell counting, neuron numbers were manually counted in two zones around the probe track (black rectangle). The single unit (SU) zone was located 0–50 µm from the probe track (blue rounded rectangle), while the control zone was 50–100 µm away from the track (red rounded rectangle). Scale bar = 100 µm. Neuron density measured in the SU zone was normalized to the neuron density in the control zone. (**f**) Scatter plot showing a strong correlation between the single unit yield and the normalized neuron density in the SU zone.

To investigate the anatomical causes behind the difference in the signal quality related to the insertion speed, we performed experiments with a single probe insertion, done either with the slowest (0.002 mm/s, n = 4 penetrations) or with the fastest (1 mm/s, n = 4 penetrations) speed. After the neuron-specific staining with NeuN, the tissue damage was evaluated as the number of NeuN-labeled neurons in the vicinity of probe track (Fig. 10). Neurons surviving within the first ~50 µm of the recording sites are the good candidates for separable single units since they usually fire spikes with amplitudes over 60 µV^1^. Thus, the extent of neuron loss in first ~50 µm band from the probe track might be a good indicator of the quality of the recorded signals. Based on the horizontal NeuN-stained brain sections, a larger cell-sparse area was observed around the probe track after faster insertions compared with slower insertions, suggesting a more extensive tissue injury in the former case (Fig. 10a,b). To quantify this observation, automatic image analysis was performed on the brain sections during which the neuron numbers were calculated in regions of interest located at different distances from the probe track (Fig. 10c; see the Methods section for more details). We found that more neurons survived the probe insertion in the first 60 µm distance from the probe when the slowest speed was used (Fig. 10d). The difference in the normalized neuron density between the two insertion speeds was statistically significant in the 0–40 µm band from the probe track (Student’s t-test; 0–20 µm band, p = 5.3 × 10^−4^; 20–40 µm band, p = 8.4 × 10^−5^). To complement the automatic cell counting, we manually counted the number of surviving neurons in the 0–50 µm zone from the probe track (single unit (SU) zone), then compared the neuron density in this zone to the neuron density in a control zone, which was located 50–100 µm far from the probe track (Fig. 10e; see the Methods section for more details). The ratio of the neuron density between the two zones was significantly different between the two speeds (0.002 mm/s vs. 1 mm/s; 98.62 ± 18.66% vs. 76.8 ± 7.39%; Student’s t-test; p = 0.016), fewer neurons were found in the SU zone after faster insertions. Furthermore, we also found a strong positive correlation (Pearson’s correlation; r = 0.79) between the single unit yield and the change of the neuron density in the SU zone compared with the control zone (Fig. 10f). Faster insertions were usually accompanied by a lower number of single units and a higher loss of neurons, while slow insertions showed a higher unit yield and an increase of neuron density in the inner zone, presumably due to tissue compression. Further details on the quality of these recordings can be found in Supplementary Fig. S2. In summary, after slower insertions, more surviving neurons were found within the critical 50 µm distance from the probe which might account for the observed better quality of neuronal recordings.

## Discussion

In this work, we have demonstrated that a slow insertion speed used for probe implantation significantly improves the quality of the recorded neuronal data acutely, for the short post implantation period of 45 minutes. *In vivo* recordings obtained from the rat neocortex after implanting high-density silicon probes with an insertion speed of 2 µm/s generally showed a higher and more stable signal-to-noise ratio compared with SNR levels calculated from data acquired after faster implantations. Furthermore, more single units were isolated after slow insertions, and the isolated neuron clusters were of higher quality as well. Additionally, a significant fraction of these neurons started to fire earlier after implantation compared with neurons recorded after faster insertions presumably indicating a more stable spiking activity after slow insertions. Based on histological analysis of the tissue injury related to the probe insertion, a significantly higher number of neurons survived the insertion in the vicinity (<50 µm) of the probe track when the slowest insertion speed was used. In conclusion, our finding suggests that the insertion speed may have a significant impact on the quality of the recorded neuronal data in acute experiments.

An increase in the SNR either indicates an increase in the signal level or a decrease in the noise level. Our experimental design allowed us to obtain data with about the same noise level among penetrations. Thus, the higher SNR measured after the slowest insertion is the result of higher signal levels, that is, neuronal action potentials with higher amplitudes were recorded (Fig. 4). As the amplitude of spikes decreases rapidly as a function of distance^29^, the occurrence of spikes with bigger amplitudes might imply that more neurons were located in the proximity of the recording probe’s microelectrodes after the slowest insertions. Consequently, more neurons close to the silicon probe may have survived the implantation procedure compared with faster insertions. This theory is supported by our results that the neuronal cell loss within the first 50 µm from the probe track was significantly lower when the slowest insertion was used compared with the fastest speed. The theory might also be supported by our observation that the ratio of single units with narrow spikes (i.e. putative interneurons) was often higher among single units obtained after slower insertions compared with the fastest insertion. Previous studies reported that 7–30% of cortical neurons may be GABAergic interneurons^28,30,31^, which is in agreement with our results. Higher interneuron ratios obtained after slower insertions suggest that these type of neurons, which are usually smaller in size compared with excitatory pyramidal cells, might be more sensitive to the insertion speed-related tissue damage.

Although the findings of this study suggest that a slow probe insertion in the range of 1 µm/s might provide better quality recordings, other studies investigating the insertion speed-related tissue damage and penetration mechanics conclude that a rapid insertion may be more favorable for neural measurements^10,15,16,18^. This contradiction might be explained by the fact that the slower end of the insertion speed assessed in previous reports was in the range between 10–125 µm/s. Thus, the effects of very slow speeds were not tested in these studies. As we have shown above (Figs 4–6), our findings were similar for the three faster insertion speeds (0.02 mm/s, 0.1 mm/s, 1 mm/s) which correspond to the speed range examined in the mentioned studies. In contrast, compared with these faster implantations, the recording quality was significantly different after the slowest insertion (0.002 mm/s). Thus, as it was also discussed by Bjornsson and colleagues^15^, a very slow probe insertion might provide the brain tissue time to recover without subsequent rupture of blood vessels. Furthermore, it may also allow accommodation of compression and stretching forces presumably resulting in the survival of a higher number of neurons next to the probe compared with faster insertions^10^.

It is important to mention that there is a difference of more than 10 minutes between the time needed to implant the probe with the slowest (insertion time: 14.17 min) and with faster speeds (insertion times: 85 s, 17 s and 1.7 s). This time difference might account for the disparities in the SNR observed between the slowest and faster speeds during the first fifteen minutes after probe insertion (Fig. 4b). That is, for the slowest speed, the time needed for the partial recovery of neuronal activity might be incorporated in the insertion time. However, the difference in the insertion time does not explain the differences found between the insertion speeds in other quantitative properties of the recorded signals (e.g. stabilized SNR level, single unit yield or cluster quality). Furthermore, there was only a slight (<15%) difference in the results when the first 15 minutes of the recordings obtained with the fastest insertion speed (corresponding to the time needed to implant the probe with the slowest speed) were omitted from the analysis (Supplementary Table S2). Therefore, a very slow insertion might have a so far unrecognized effect on the preservation of tissue integrity, and thus on the recording quality.

Although our findings suggest that a slow insertion speed might be advantageous over faster speeds, it is important to discuss some of the limitations of this study. For instance, during some of the probe insertions, we observed a noticeable brain dimpling, even when the dura mater was removed. This type of brain deformation might be problematic for the following reason. When the probe finally penetrates the brain during dimpling, it does this with a much higher speed than intended due to the sudden decrease of tension between the probe and the tissue. This relatively fast penetration might damage the tissue significantly, and, consequently, cancel the beneficial effects of slow insertion. This was especially apparent after one of the insertions carried out with the slowest speed: we could cluster only four well-isolated single units and the signal-to-noise ratio was very low, in the range observed after faster insertions. In addition, signs of bleeding were detected in layer V of the cortical tissue examined post mortem (Supplementary Fig. S3). The tip geometry (e.g. opening angle) of the probe might also have a significant effect on the tissue trauma caused by the insertion, especially during the penetration through the dura and pia mater^13^. The surface response model developed by Andrei and colleagues predicted that lowering both the opening angle of a chisel-shaped probe tip and the insertion speed decreases the insertion force and brain dimpling^13^. This, in turn, might dampen the tissue strain and stress induced during insertion. Using probes with a pointy tip instead of probes with a chisel-shaped tip might be another solution to reduce the extent of brain dimpling significantly^32^. Alternatively, removing the pia mater carefully above the implantation site may also solve this issue. However, although the use of the latter method might alleviate the deformation of the cortex during probe implantation, it increases the probability of damaging a blood vessel on the brain surface as well.

Since there is a pronounced difference in the spiking activity between cortical layers during ketamine/xylazine-induced slow waves^21,22^, variations in the penetration depth might impact the results obtained here. For example, when a large number of recording sites are located outside of layer V (from where the strongest neuronal activity can be recorded), the single unit yield obtained might be significantly lower compared when the probe records mostly from layer V. However, here we found that the insertion depth did not vary systematically with the insertion speed and most of the recording sites were located in layer V (Fig. 2). Furthermore, restricting the analysis only to single units located in layer V produced similar results (Supplementary Table S3).

It is also important to emphasize that only two types of single-shank recording probes were tested in this study. Implanting other types of probes, for instance probes with multiple shanks, different shank geometry or stiffness might produce different results; as well as testing the effect of the insertion speed in other animal species or in different brain regions. Moreover, testing very fast insertion speeds in the range of m/s, which is used to implant specific probes (e.g. Utah arrays), were out of the scope of this study as well^17,33^. Finally, we assessed the effects of the insertion speed on the recording quality only on a short timescale. Neural probes, however, are often implanted for the long term, for example to study the relationship between the observed neuronal activity and a specific animal behavior. Whether the results might be similar in case of such chronic implantations, still needs to be tested.

Most electrophysiological studies, which measure the extracellular electrical activity with neural probes implanted into the brain tissue, use an insertion speed of 1 µm/s or higher for probe implantation (Supplementary Table S1). However, only about half of the examined reports applied very slow insertion speeds (~1 µm/s). Thus, based on our findings, insertion speeds used traditionally might in many cases not lead to neuronal recordings with the best quality. To obtain high-quality recordings, several commercially available motorized insertion tools are available allowing to move neural probes with a slow speed and a fine step size. The use of such devices also eliminates micromotions of the probe which may occur during manual probe insertion when the micromanipulator is operated by hand.

Although, to gain high-quality recordings, it is possible to use implantation speeds below 1 µm/s, the time needed to implant the recording probe during acute experiments might be a limiting factor. For instance, implanting probes with an insertion speed of 1 µm/s into deep layers of the rat neocortex might take about half an hour. Such a relatively long time devoted only to probe implantation might be not practical for many experiments, especially for those targeting deeper brain structures. Thus, to obtain high-quality neuronal activity from brain structures located deeper than 1–2 mm in the tissue, we suggest an alternative implantation strategy. The insertion of the probe might be started with a higher speed (~0.1 mm/s), then, when we are only 1–2 mm away from the targeted brain structure, we can change to very slow insertion speeds in the range of 1 µm/s. Using this strategy saves time during implantation but may still provide high-quality recordings. In case there is no option for a slow insertion during the experiment, based on the rapid change in the SNR after faster implantations (Fig. 4b), we advise to wait at least 15–30 minutes for the stabilization of signals before starting to record the neuronal activity. This way the brain tissue has time to partially recover after the acute injury caused by the probe insertion. However, if the necessary equipment is available, we suggest implanting probes as slowly as possible, since, on average, the single unit yield was more than two times higher after the slowest insertion compared with that obtained after the fastest insertion (Fig. 5a). Thus, as a positive effect, using very slow insertion speeds might considerably reduce the number of animals necessary for an acute electrophysiology study by measuring the activity of a higher number of neurons during a single experiment.

## Methods

### Animal surgery

All experiments were performed according to the EC Council Directive of September 22, 2010 (2010/63/EU), and all procedures were reviewed and approved by the Animal Care Committee of the Research Centre for Natural Sciences of the Hungarian Academy of Sciences and by the National Food Chain Safety Office of Hungary (license number: PEI/001/2290-11/2015). Wistar rats (n = 23; 291 ± 48 g; gender balanced) were anesthetized with a mixture of ketamine (75 mg/kg of body weight) and xylazine (10 mg/kg of b.w.) injected intramuscularly. If necessary, supplementary ketamine/xylazine injections were given to maintain the depth of anesthesia during surgery and recordings. The animals were placed in a stereotaxic frame (David Kopf Instruments, Tujunga, CA, USA) after they reached the level of surgical anesthesia. The body temperature of rats was maintained with a homeothermic heating pad connected to a temperature controller (Supertech, Pécs, Hungary). After the removal of the skin and the connective tissue from the top of the skull, a circular craniotomy with a diameter of 3 mm was drilled over both brain hemispheres (Fig. 1a; center of the craniotomy: anterior-posterior (AP): –3 mm; medial-lateral (ML): 3 mm; with respect to the bregma^34^). Then, to avoid excessive brain dimpling during the insertion of single-shank silicon-based probes^2^, a small slit was carefully made in the dura mater above the trunk region of the primary somatosensory cortex (S1Tr) using a 30-gauge needle. For a post-mortem histological verification of the recording location of the probe^35^, the backside of the silicon shank was coated with red-fluorescent dye 1,1-dioctadecyl-3,3,3,3-tetramethylindocarbocyanine perchlorate (DiI, D-282, ~10% in ethanol, Thermo Fischer Scientific, Waltham, MA, USA) before insertion. After that, the silicon probe mounted on a motorized stereotaxic micromanipulator (Robot Stereotaxic, Neurostar, Tübingen, Germany) was driven into the brain tissue with the selected insertion speed to a dorsoventral brain depth of 1700 µm (Fig. 1b; details of the insertion protocol are described in the next section). During probe insertion, care was taken to avoid damaging blood vessels located on the brain surface. Room temperature physiological saline solution was regularly dropped into the cavity of the craniotomy to prevent dehydration of the neocortex. A stainless steel needle inserted in the neck muscle of the animal served as the reference electrode during recordings.

### Experimental protocol

Two types of single-shank silicon probes were used in the study. The first probe type has a 50-µm-thick shank which contains 128 closely-packed recordings sites^2^. During each experiment with this probe type (n = 10 rats), four penetrations were carried out, each with a different speed (0.002 mm/s, 0.02 mm/s, 0.1 mm/s, 1 mm/s). The sequence of the insertion speeds was chosen quasi-randomly (Supplementary Table S4). Two penetrations have been made in each brain hemisphere (Fig. 1a), then, before the third penetration, the recording probe was cleaned and repainted with DiI (see details of the cleaning procedure in the next section). During a probe insertion, neurons as far as 100 µm from the probe might be injured or killed^36^. Therefore, to avoid recording from the proximity of brain tissue damaged during the previous insertion, penetration sites were at least 500 µm far from each other. For the second penetration in the same hemisphere, the probe was moved in most cases only along the mediolateral axis to its new position. However, if the new penetration site was blocked by large blood vessels located on the top of the cortex, the probe was moved a few hundred micrometers along the anteroposterior axis to a more anterior or posterior location. Recording of the neuronal activity started immediately after the tip of the silicon probe reached the appropriate cortical depth (1.7 mm from the top of the cortex; Fig. 1b,c). For each insertion speed, the travel time of the probe from the surface of the brain to the specified recording depth was the following: 0.002 mm/s–850 s (14.17 min), 0.02 mm/s–85 s, 0.1 mm/s –17 s, 1 mm/s–1.7 s. The step size of the motorized micromanipulator used for implantation (Robot Stereotaxic, Neurostar) was ~1.4 µm. After recording, probes were retracted using the fastest speed (1 mm/s).

The other, thinner probe used was a commercially available, 32-channel probe with a 15-µm-thick shank (NeuroNexus A1x32-5 mm-25-177). During each experiment with this probe type (n = 5 rats), four penetrations were carried out, two insertions with the slowest (0.002 mm/s) and two with the fastest (1 mm/s) insertion speed (Supplementary Table S5). The experimental protocol was the same as for the 128-channel probe described above.

To investigate the extent of tissue damage in the vicinity of the penetration, the 128-channel probe was used in another set of experiments (n = 8 rats). In each rat, the probe was inserted only once, into the left brain hemisphere and either with the slowest (0.002 mm/s, n = 4 penetrations) or with the fastest (1 mm/s; n = 4 penetrations) speed. The experimental protocol was the same as described above, except that at the end of the experiment the probe was retracted with the slowest speed to reduce the tissue damage related to the removal of the probe.

### Electrophysiological recordings

Spontaneously occurring brain electrical activity of all animals was recorded either on 128 channels (in the case of the 128-channel probe) or on 32-channels (in the case of the 32-channel probe) using an Intan RHD-2000 electrophysiological recording system (Intan Technologies, Los Angeles, CA, USA). One 64-channel and two 32-channel amplifier boards were used in the case of the 128-channel probe, while a single 32-channel board was used with the 32-channel probe. The recording system was connected to a laptop via USB 2.0. Wideband signals (0.1–7500 Hz) were recorded with a sampling frequency of 20 kHz/channel and a resolution of 16 bit. Data files containing five-minute-long continuous recordings were saved to the hard drive of the laptop for offline data analysis. Forty-five minutes of neuronal data (i.e. nine data files) were collected after each penetration. Due to a technical error, no measurements could be obtained for one animal after inserting the 128-channel probe with 1 mm/s. After the second penetration, as well as at the end of the experiment, the probe was withdrawn and cleaned by immersing it into 1% Tergazyme solution (Sigma-Aldrich, St. Louis, MO, USA) for at least 30 minutes followed by rinsing with distilled water for 2 minutes. Two identical 128-channel probes from the same manufacturing batch were used during the experiments^2^, each probe was implanted in five rats (n = 20 penetrations/probe). The probes contained a maximum of two unfunctional recording sites. In the second set of experiments (thin probe; n = 20 penetrations), a single 32-channel probe was used with all its recording sites functional. In the third set of experiments (assessment of tissue damage; n = 8 penetrations), a single 128-channel probe without unfunctional electrodes was used for the insertions.

### Verification of the recording location

To detect the tracks of the silicon probe in the brain tissue, we used a histological procedure described previously^2^. In short, the animal was deeply anesthetized after the experiment, then transcardially perfused with physiological saline solution (100 ml) followed by a fixative containing 4% paraformaldehyde in 0.1 M phosphate buffer (PB, pH = 7.4, 250 ml). The fixed brain was removed from the skull and stored at 4 °C overnight in the fixative solution. After that, 60-µm-thick coronal sections were cut with a vibratome (Leica VT1200, Leica Microsystems, Wetzlar, Germany). Following washing in 0.1 M PB, brain sections were mounted from gelatin onto microscopic slides and air dried. To identify brain sections containing fluorescent marks of DiI corresponding to the probe track (Fig. 1c), the slides were examined under a light microscope (Leica DM2500, Leica Microsystems) equipped with a fluorescence LED illumination source (SFL4000, Leica) and with a digital camera (DP73, Olympus, Tokyo, Japan). After that, the brain sections were processed for cresyl violet (Nissl) staining, dehydrated in xylene and coverslipped with DePex (SERVA Electrophoresis, Heidelberg, Germany). Finally, to verify the recording location based on the stereotaxic rat brain atlas^34^, Nissl-stained sections containing the track of the silicon probe were photographed under the microscope (Fig. 1c).

### Visualization of neurons around the probe track using NeuN immunostaining

The impact of the probe insertion speed on the neuronal survival near the implant was studied in 8 animals by immunostaining for NeuN, a nuclear antigen found only in neuronal cells. After the *in vivo* experiments, the brain of the animals was fixed and removed in the same manner as described in the section above. The removed brain was postfixed in the same fixative solution overnight at 4 °C. On the following day, 60 µm thick horizontal sections were cut with a vibratome. First, the probe track was identified on the unstained sections. The fluorescent marks of DiI enabled the determination of the accurate position of the track of the probe in the brain tissue. Sections were mounted from PB and examined in the aforementioned Leica DM2500 microscope using filters for Cy3 (exciting filter bandpass 530–560). The identified track was photographed in all sections. Subsequently, the still wet sections were placed back to PB and processed for immunohistochemistry using a protocol described previously^37^. Briefly, after blocking the endogenous peroxidase activity and the nonspecific immunoglobulin binding of the tissue, a monoclonal mouse antibody against neuronal nuclei (NeuN, EMD Millipore, Billerica, MA, USA, 1:2000) was applied for visualization of the neuronal cell bodies (2 days, 4 °C). This was followed by the incubation in biotinylated anti-mouse immunoglobulin G (Vector Laboratories, Burlingame, CA, USA; 1:250, 2 h), then the incubation in avidin-biotinylated horseradish peroxidase complex (Vector Laboratories; 1:250, 1.5 h). Sections were preincubated in 3,3′-diaminobenzidine-tetrahydrochloride hydrate chromogen (0.05%) and developed by 0.01% H_2_O_2_. The immunostained sections were mounted, dehydrated in xylene and cover-slipped with DePex.

### Quantification of neuronal cell loss around the probe track

To study the effect of the insertion speed on the neuronal survival near the probe, the density of neurons surrounding the track was analyzed with quantitative methods. The immunostained samples were examined at the light microscopic level. In the NeuN-immunolabelled sections, the probe tracks were easily identifiable with the help of the DiI photographs of the same sections. For each animal, every sixth section (starting from the pial surface of the brain towards the white matter) was immunostained and analyzed. This corresponds to the following approximate neocortical depths: 420–480 µm, 780–840 µm and 1140–1200 µm. Twenty-three sections were evaluated altogether, deriving from 8 animals (0.002 mm/s, 11 sections; 1 mm/s, 12 sections). The probe tracks were photographed at 10- and 20-fold magnifications.

The quantitative analysis of the NeuN-immunostained sections was performed both by manual counting (using the 20-fold magnification) and by an automated method developed in MATLAB (MathWorks, Natick, MA, USA) environment (using the 10-fold magnification).

In the case of the automated method, a 100 µm long and 10 µm wide rectangle (parallel to the previous placement of the probe) was defined manually with the center of the track located in the middle of the rectangle (Fig. 10c). Note that the size of the track does not reflect the real size of the cross-section of the probe (100 × 50 µm^2^), since during its removal, the brain tissue closed beneath it. Then, the neuron density was examined in the function of the distance from the implant track, as follows. From the manually selected outline (probe track, 0 µm), the number of NeuN-positive cell bodies were counted, and density values were calculated by the MATLAB routine in 20 µm wide and 100 µm long bins, up to 400 µm on the two long sides of the track (Fig. 10c). Finally, the density values in the first 10 bins (0–200 µm) were normalized to the average values of the 160 to 400 µm region.

To complement the results of the automatic cell counting, neuron numbers were manually determined in two zones around the probe track (Fig. 10e). The single unit (SU) zone was the first 50 µm from the probe track, while the control zone was the region 50–100 µm far from the track. From the neuron numbers obtained, the density of neurons (neuron/mm^2^) was calculated. Then, the ratio of the neuron density between the two zones was computed to assess the neuron loss in the SU zone. For each experiment, the calculations were done for two brain sections located in the level of the recording sites (780–840 µm and 1140–1200 µm).

### Calculation of the signal-to-noise ratio

The signal-to-noise ratio (SNR) of cortical recordings was calculated in MATLAB with a method described previously^2,37,38^. First, the wideband data was filtered between 500 and 5000 Hz (band-pass filter; zero-phase shift; 24 dB/octave) to remove lower frequencies corresponding to local field potentials. After that, we applied the nonlinear energy detector (NEO) method on the filtered traces to detect neuronal spikes for the calculation of the signal power. For each penetration, the SNR was calculated from consecutive, 30-second-long sections of recordings, separately on each recording channel (n = 90 SNR values/channel), using the following formula:

$$SN{R}_{dB}=20\cdot lo{g}_{10}\frac{\frac{1}{N}{\sum }_{n=1}^{N}\,RMS(spik{e}_{n}(t))}{{\hat{\sigma }}_{noise}}$$

where RMS(spike_n_(t)) is the root mean square of spike *n* measured in a 1-ms-long window centered around the spike detected with the NEO method^38^. The level of noise (${\hat{\sigma }}_{noise}$) was estimated from the noise standard deviation (SD) which was calculated by the root mean square of all mean-centered values outside the spike windows^38^. Then, for each 30-second-long data segment, we calculated the average and SD of the SNR across channels. SNR values computed from segments of recorded data containing large (>1 mV) spike artifacts or high-frequency noise contaminating the unit activity were removed from the analysis. Furthermore, a faster brain activity (delta waves with a characteristic frequency of 2–4 Hz) might appear in light anesthesia, during which neuronal activity in the neocortex is highly synchronous. Based on our observations, recording segments containing delta waves showed a considerably higher SNR than measured during ketamine/xylazine-induced slow-wave activity, irrespective of the insertion speed used. Therefore, to avoid the bias arising from this difference, SNR values computed in sections of recordings which included delta waves were excluded from further analysis as well. Finally, to examine the change of the SNR over time, the grand average SNR was computed across insertions carried out with the same speed, then visualized for each insertion speed.

### Spike sorting and calculation of single unit properties

To assign the recorded spikes to individual neurons, automatic spike sorting was performed using a software (Kilosort) developed in MATLAB^39^. Manual revision of the single unit clusters detected by Kilosort was done in Phy, an open source neurophysiological data analysis package written in Python (https://github.com/kwikteam/phy). After the revision of spike sorting results, wideband spikes of each single unit cluster were averaged together to obtain the average spike waveforms. Only well-isolated single units were used for further analysis. The selection of well-isolated units was aided by using two criteria. These criteria allowed us to exclude low quality units as well as to decrease the effect of subjective decisions of the operator during the manual curation of neuron clusters. We defined a single unit as well isolated if it had a clear refractory period, as well as a spike waveform with a peak-to-peak amplitude over 60 µV which is large enough for a reliable spike sorting^1^. The peak-to-peak amplitude was defined as the amplitude difference between the negative peak (or trough) and the largest positive peak of the average spike waveform. Since the spikes of the same neuron were usually recorded by multiple, adjacent recording sites^2^, the peak-to-peak amplitude was computed only on that recording channel on which the spike waveform of the particular single unit appeared with the largest amplitude. Furthermore, a clear refractory period was defined by a “violation rate” below 2%. The violation rate of a single unit cluster (expressed in percentage) is the number of spikes that are followed within 2 ms by other spikes belonging to the cluster divided by the total number of spikes in that particular cluster. The number of units excluded for each insertion speed are shown in Supplementary Tables S6 and S7. It is important to note that including these excluded units in the analysis would not affect our findings significantly. Only a low percent of the units were excluded from the analysis which, based on our criteria, usually had a low quality (low peak-to-peak amplitude and isolation distance).

We also determined the latency of the first spike fired by well-isolated neurons, that is, when these started firing after probe insertion. The peak-to-peak amplitude, violation rate and first spike latency were calculated using custom-written MATLAB scripts. Additionally, the isolation distance corresponding to well-separated single units was calculated to compare the quality of clusters across different insertion speeds (https://github.com/cortex-lab/sortingQuality)^26^. To differentiate between single units with wide and narrow spikes (corresponding to putative principal cells and interneurons, respectively) we computed the duration (trough-to-peak time) of each spike waveform^28^ (Fig. 7a). The distribution of trough-to-peak times was significantly bimodal (Hartigan’s dip test^40^; p = 6.942 × 10^−6^). Single units with spike waveforms having a trough-to-peak time below 0.6 ms were classified as putative interneurons (narrow spikes), while remaining neuron clusters were considered as putative principal cells (wide spikes). Five neurons were not classified because these did not have typical spike waveforms. Finally, we calculated the ratio of putative interneurons and principal cells for each insertion speed (Fig. 7b).

### Statistical analysis

One-way ANOVA was used to examine the variation in the insertion depth across insertion speeds (Fig. 2) and two-tailed Student’s t-test was used for the assessment of the neuronal cell loss (Fig. 10). None of the other variables of interest exhibited normal distribution according to the Kolmogorov-Smirnov and Shapiro-Wilk tests. Therefore, for the data obtained from the 128-channel recordings (four insertion speeds), non-parametric Kruskal-Wallis test was used for all variables, except for the putative interneuron and principal cell numbers where we used chi-square test. When a significant difference was found between insertion speeds by the Kruskal-Wallis test, post-hoc analysis was performed for all pairwise comparisons using Dunn’s test with Bonferroni correction. For the data acquired from the 32-channel recordings (two insertion speeds), non-parametric Mann-Whitney U test was used for all variables. p values < 0.05 were considered significant. Box-and-whisker plots (Figs 2, 4–6 and 9) showing the distribution of data are presented as follows. The middle line indicates the median, while the boxes correspond to the 25^th^ and 75^th^ percentile. Whiskers mark the minimum and maximum values. The average is depicted with a black dot.

## Electronic supplementary material

Supplementary Material

## Data Availability

The datasets generated during and/or analyzed during the current study are available from the corresponding author on reasonable request.
