# Supplementary material for: Slow insertion of silicon probes improves the quality of acute neuronal recordings
Source: Sci Rep. 2019 Jan 14;9:111. doi: 10.1038/s41598-018-36816-z (PMC6331571; doi:10.1038/s41598-018-36816-z)
Supplement: Supplementary file 1 — Supplementary Material [file 41598_2018_36816_MOESM1_ESM.pdf]

Supplementary Material

Slow insertion of silicon probes improves the quality of acute neuronal recordings

Richárd Fiáth[1,2], Adrienn Lilla Márton[2], Ferenc Mátyás[1,3], Domonkos Pinke[4], Gergely Márton[1,2], Kinga Tóth[1], István Ulbert[1,2]

[1] Institute of Cognitive Neuroscience and Psychology, Research Centre for Natural Sciences, Hungarian Academy of Sciences, Magyar tudósok körútja 2, H-1117 Budapest, Hungary

[2] Faculty of Information Technology and Bionics, Pázmány Péter Catholic University, Práter utca 50/A, H-1083 Budapest, Hungary

[3] Department of Anatomy and Histology, University of Veterinary Medicine, István utca 2, H-1078, Budapest, Hungary

[4] Institute of Experimental Medicine, Hungarian Academy of Sciences, Szigony utca 43, H-1083 Budapest, Hungary

Contents:

- **Supplementary Table S1**
- **Bibliographic details of studies listed in Supplementary Table S1**
- **Supplementary Table S2**
- **Supplementary Table S3**
- **Supplementary Table S4**
- **Supplementary Table S5**
- **Supplementary Table S6**
- **Supplementary Table S7**
- **Supplementary Figure S1**
- **Supplementary Figure S2**
- **Supplementary Figure S3**

Supplementary Table S1 – Example electrophysiological studies listed in the order of the insertion speed used for probe implantation.

	Study	Insertion speed (mm/s)	Insertion speed (reported)	Animal species	Acute/ Chronic	Extracellular recording device
1	Moxon et al., 2004	0.0002	10 µm/min	rat	C	ceramic insulated, thin-film multisite electrodes
2	Kisley and Gerstein, 1999	0.0002	~10 µm/min	rat	A / C	single wire electrodes/tetrodes
3	Ward et al., 2009	0.0002	~10 µm/min	rat	C	thin-film ceramic-based microelectrode array
4	Bardy et al., 2006	0.0003 - 0.001	20 - 60 µm/min	cat	A	stainless steel microelectrodes
5	Huang et al., 2017	0.0003 - 0.001	20 - 60 µm/min	cat	A	stainless steel microelectrodes
6	Thimm and Funke, 2015	0.0003	~20 µm/min	rat	A	bundle of three tungsten electrodes
7	Wiebe and Staubli, 1999	0.0004	~25 µm/min	rat	C	array of Teflon-coated, stainless steel microwires
8	Cardoso-Cruz et al., 2013	0.0008	50 µm/min	rat	C	array of isonel-coated tungsten microwires
9	Devilbiss et al., 2006	0.0008	~50 µm/min	rat	C	bundles of Teflon-coated stainless steel microwires
10	Devilbiss and Waterhouse, 2011	0.0008	~50 µm/min	rat	C	microwire bundle
11	Lasztoczi and Klausberger, 2016	0.0008 - 0.0017	50 - 100 µm/min	mouse	A	multi-shank silicon probes
12	Chung et al., 2017	0.0008 / 0.0017	50 / 100 µm/min	mouse	A / C	Buzsaki-type silicon probes
13	Neto et al., 2016	0.001	1 µm/s	rat	A	high-density silicon polytrodes
14	Mechler et al., 2011	0.001	~1 µm/s	monkey,cat	A	tetrodes
15	Kondabolu et al., 2016	0.001 - 0.002	1 - 2 µm/s	mouse	A	borosilicate glass electrode/laminar silicon probes
16	Lim et al., 2016	0.001 - 0.002	1 - 2 µm/s	songbird	A	four-shank silicon probes
17	Suyatin et al., 2013	0.001 - 0.01	1 - 10 µm/s	rat	A	nanowire-based electrode
18	Han et al., 2009	0.0015	1.5 µm/s	monkey	A	tungsten microelectrodes
19	Maris et al., 2013	0.0015	1.5 µm/s	monkey	C	tungsten microelectrodes

	Study	Insertion speed (mm/s)	Insertion speed (reported)	Animal species	Acute/ Chronic	Extracellular recording device
20	Musall et al., 2017	0.0017	100 µm/min	rat	A / C	linear silicon probes
21	Wang et al., 2012	0.0017	100 µm/min	rat	C	silicon-based multielectrode array
22	Schoenfeld et al., 2014	0.0017	100 µm/min	mouse	C	stainless steel electrodes
23	Venkatachalam et al., 1999	0.0017	100 µm/min	rat	C	parylene-coated tungsten microelectrodes
24	Crist and Lebedev, 2008	0.0017	100 µm/min	monkey	C	microelectrode arrays
25	Fontanini and Katz, 2005	0.0017	100 µm/min	rat	C	microwire bundles
26	Wiest et al., 2008	0.0017	≤100 µm/min	rat	C	array of tungsten microelectrodes
27	Denman et al., 2017	0.0017	~100 µm/min	mouse	A	high-density planar silicon electrode arrays
28	Nicolelis et al., 1997	0.0017	~100 µm/min	rat	C	array of Teflon-coated, stainless steel microwires
29	Nicolelis et al., 2003	0.0017	~100 µm/min	monkey	C	insulated stainless steel/tungsten microwire arrays
30	Prasad et al., 2014	0.0017	~0.1 mm/min	rat	C	16-site floating microelectrode array
31	Oliveira-Maia et al., 2008	0.0017 - 0.0033	100 - 200 µm/min	mouse, rat	C	array of tungsten microelectrodes
32	Li et al. 2018	0.002	2 µm/s	mouse	A	32-channel silicon probes
33	Stolzberg et al., 2012	0.002	2 µm/s	rat	A	linear silicon probes
34	McAlinden et al., 2015	0.002	~2 µm/s	mouse	A	32-channel linear silicon-based optrodes
35	Raducanu et al., 2017	0.002	~2 µm/s	rat	A	silicon-based CMOS probes
36	Scharf et al., 2016	0.002	~2 µm/s	mouse	A	32-channel linear silicon-based optrodes
37	Kayser et al., 2015	0.002	<2 µm/s	rat	A	multi-shank silicon-based tetrode probes
38	Sakata, 2016	0.002	≤2 µm/s	rat	A	single-shank silicon probes
39	Okun et al., 2016	0.002 - 0.004	2 - 4 µm/s	mouse	C	multi-shank silicon-based tetrode probes

	Study	Insertion speed (mm/s)	Insertion speed (reported)	Animal species	Acute/ Chronic	Extracellular recording device
40	Chandrasekaran et al., 2017	0.002 - 0.005	~2 - 5 µm/s	monkey	A	linear multi-contact electrodes (U-probe)
41	Yamamoto and Wilson, 2008	0.002 / 0.05	~2 µm/s / ~50 µm/s	rat	C	tetrodes made from polyimide-coated nichrome wires
42	O'Shea and Shenoy, 2018	0.003	3 µm/s	monkey	C	linear electrode array (V-probe)
43	Guo et al., 2014	0.003	~3 µm/s	mouse	A	single-shank or multi-shank silicon probes
44	Scherberger et al., 2003	0.003	0.2 mm/min	monkey	C	array of Parelene-C insulated tungsten microwires
45	Shiramatsu et al., 2016	0.003 - 0.004	3 - 4 µm/s	rat	A	multi-shank silicon probes
46	Bray et al., 2016	0.005	5 µm/s	rat	A	action potential-oxygen (APOX) electrodes
47	Scott et al., 2012	0.01	<10 µm/s	mouse	A	multisite silicon probes
48	Du et al., 2011	0.01	≤10 µm/s	mouse	C	multisite silicon probes
49	Mols et al., 2017	0.01	10 µm/s	mouse	C	multisite silicon probes
50	Paralikar and Clement, 2008	0.01	10 µm/s	rat	A / C	array of tungsten microwires insulated with polyimide
51	Zhang et al., 2018	0.01	10 µm/s	monkey	A	silicon-based dual-mode microelectrode array
52	Zhao et al., 2016	0.01	10 µm/s	rat	A	dual-sided silicon-based microelectrode array
53	Etemadi et al., 2016	0.01 / 0.1	10 µm/s / 100 µm/s	rat	C	bundles of parylene C coated platinum electrodes
54	Leiser and Moxon, 2006	0.01 / 0.1	10 µm/s / ~100 µm/s	rat	A	epoxylite-insulated tungsten microelectrodes
55	Yang et al., 2016	0.01 / 0.05 - 0.1	10 µm/s / 50 - 100 µm/s	mouse	A	tungsten microelectrodes
56	Dryg et al., 2015	0.016	1 mm/min	rat	C	stainless steel microwires (PlasticsOne)

	Study	Insertion speed (mm/s)	Insertion speed (reported)	Animal species	Acute/ Chronic	Extracellular recording device
57	Hampson et al., 2004	0.016	1 - 2 mm/min	monkey	A	tungsten microwires
58	McGinty and Grace, 2008	0.016	≤1 mm/min	rat	A	borosilicate glass electrodes
59	Godlove et al., 2014	0.025	25 μm/s	monkey	A	Teflon-coated tungsten microelectrodes
60	Agorelius et al., 2015	0.05	50 μm/s	rat	C	3D flexible electrode array
61	Deku et al., 2018	0.05	50 μm/s	rat	A	amorphous silicon carbide microelectrode array
62	Sawahata et al., 2016	0.05	~50 μm/s	mouse	A	fine silicon wire electrodes
63	Zhang et al., 2015	0.08 - 0.1	80 - 100 μm/s	rat	A	silicon probe
64	Lee et al., 2012	0.1	100 μm/s	rat	A	flexible liquid crystal polymer (LCP) neural probes
65	Ramrath et al., 2009	0.1	0.1 mm/s	rat	A	bipolar microelectrodes
66	Seidl et al., 2012	0.1	0.1 mm/s	rat	A	CMOS-based silicon microprobe arrays
67	Raducanu et al., 2017	0.1	~0.1 mm/s	rat	A	silicon-based CMOS probes
68	Felix et al., 2013	0.13 - 0.5	0.13 - 0.5 mm/s	rat	C	thin-film polymer probes
69	Shen et al., 2015	0.5	500 μm/s	rat	C	extracellular matrix-based intracortical microelectrodes
70	Johnson et al., 2008	0.5 - 1.5	0.5 - 1.5 mm/s	rat	A	linear silicon probes
71	Han et al., 2012	1	1 mm/s	cat	C	silicon-based multisite microelectrode arrays
72	Jackson and Fetz, 2007	1	~1 mm/s	monkey	C	Teflon-insulated tungsten microwire array
73	Kozai et al., 2015a	1	~1 mm/s	mouse	C	single-shank planar silicon probes
74	Rohatgi et al., 2009	1.2	1.2 mm/s	rat	A	Michigen-type silicon probes
75	Escamilla-Mackert et al., 2009	1.2	1.2 mm/s	rat	A	single- and multi-shank silicon probes

	Study	Insertion speed (mm/s)	Insertion speed (reported)	Animal species	Acute/ Chronic	Extracellular recording device
76	Zeitler et al., 2006	1.5	1.5 mm/s	monkey	A	tungsten microelectrodes
77	Kozai et al., 2015b	2	2 mm/s	mouse	C	single-shank Michigan-type silicon probes
78	Kozai et al., 2016	2	~2 mm/s	mouse	C	double-shank silicon probes
79	Lee et al., 2014	20	20 mm/s	rat	C	silicon-based planar microelectrode arrays
80	Lee et al., 2017	20	20 mm/s	rat	C	silicon-based planar microelectrode arrays
81	Bai et al., 2000	200 - 1000	20 - 100 cm/s	guinea pig	A	three-dimensional silicon microelectrode arrays
82	Han et al., 2012	1000	1 m/s	cat	C	silicon-based multisite microelectrode arrays
83	Rennaker et al., 2005	1490	1.49 m/s	rat	C	array of tungsten microelectrodes
84	Barrese et al., 2013	8000 - 10000	8 - 10 m/s	monkey	C	silicon-based microelectrode array (Utah)
85	Barrese et al., 2016	8000 - 10000	8 - 10 m/s	monkey	C	silicon-based microelectrode array (Utah)
86	Ward et al., 2009	8300	≥8.3 m/s	rat	C	silicon-based microelectrode array (Utah)
87	Ward et al., 2009	8300	≥8.3 m/s	rat	C	iridium oxide microelectrode array (Utah)
88	Dryg et al., 2015	27800	~27,8 m/s	rat	C	Pt-Fe microwires

Bibliographic details of studies listed in Supplementary Table S1

Agorelius J, Tsanakalis F, Friberg A, Thorbergsson PT, Pettersson LM and Schouenborg J (2015). "An array of highly flexible electrodes with a tailored configuration locked by gelatin during implantation-initial evaluation in cortex cerebri of awake rats." Front Neurosci **9**: 331.

Ashby CR, Minabe Y, Stemp G, Hagan JJ and Middlemiss DN (2000). "Acute and Chronic Administration of the Selective D3Receptor Antagonist SB-277011-A Alters Activity of Midbrain Dopamine Neurons in Rats: An In Vivo Electrophysiological." J Pharmacol Exp Ther **294** (3): 1166-1174.

Bai Q, Wise KD and Anderson DJ (2000). "A high-yield microassembly structure for three-dimensional microelectrode arrays." IEEE Trans Biomed Eng **47**(3): 281-289.

Bardy C, Huang JY, Wang C, FitzGibbon T and Dreher B (2006). "'Simplification' of responses of complex cells in cat striate cortex: suppressive surrounds and 'feedback' inactivation." J Physiol **574**(Pt 3): 731-750.

Barrese JC, Aceros J and Donoghue JP (2016). "Scanning electron microscopy of chronically implanted intracortical microelectrode arrays in non-human primates." J Neural Eng **13**(2): 026003.

Barrese JC, Rao N, Paroo K, Triebwasser C, Vargas-Irwin C, Franquemont L and Donoghue JP (2013). "Failure mode analysis of silicon-based intracortical microelectrode arrays in non-human primates." J Neural Eng **10**(6): 066014.

Bray N, Burrows FE, Jones M, Berwick J, Allan SM and Schiessl I (2016). "Decreased haemodynamic response and decoupling of cortical gamma-band activity and tissue oxygen perfusion after striatal interleukin-1 injection." J Neuroinflammation **13**(1): 195.

Cardoso-Cruz H, Lima D and Galhardo V (2013). "Impaired Spatial Memory Performance in a Rat Model of Hippocampus–Prefrontal Cortex Connectivity." J Neurosci **33**(6):2465-2480.

Chandrasekaran C, Peixoto D, Newsome WT and Shenoy KV (2017). "Laminar differences in decision-related neural activity in dorsal premotor cortex." Nat Commun **8**(1): 614.

Chung J, Sharif F, Jung D, Kim S and Royer S (2017). "Micro-drive and headgear for chronic implant and recovery of optoelectronic probes." Sci Rep **7**(1): 2773.

Crist RE and Lebedev MA. "Multielectrode Recording in Behaving Monkeys." In: Nicolelis MAL, (editor), “Methods for Neural Ensemble Recordings." 2nd edition. Boca Raton (FL): CRC Press/Taylor & Francis, 2008. Chapter 9.

Deku F, Cohen Y, Joshi-Imre A, Kanneganti A, Gardner TJ and Cogan SF (2018). "Amorphous silicon carbide ultramicroelectrode arrays for neural stimulation and recording." J Neural Eng **15**(1): 016007.

Denman DJ, Siegle JH, Koch C, Reid RC and Blanche TJ (2017). "Spatial Organization of Chromatic Pathways in the Mouse Dorsal Lateral Geniculate Nucleus." J Neurosci **37**(5): 1102-1116.

Devilbiss DM and Waterhouse BD (2011). "Phasic and tonic patterns of locus coeruleus output differentially modulate sensory network function in the awake rat." J Neurophysiol **105**(1): 69-87.

Devilbiss DM, Page ME and Waterhouse BD (2006). "Locus ceruleus regulates sensory encoding by neurons and networks in waking animals." J Neurosci **26**(39): 9860-9872.

Dryg ID, Ward MP, Qing KY, Mei H, Schaffer JE and Irazoqui PP (2015). "Magnetically Inserted Neural Electrodes: Tissue Response and Functional Lifetime." IEEE Trans Neural Syst Rehabil Eng **23**(4): 562-571.

Du J, Blanche TJ, Harrison RR, Lester HA and Masmanidis SC (2011). "Multiplexed, high density electrophysiology with nanofabricated neural probes." PLoS One **6**(10): e26204.

Etemadi L, Mohammed M, Thorbergsson PT, Ekstrand J, Friberg A, Granmo M, Pettersson LM and Schouenborg J (2016). "Embedded Ultrathin Cluster Electrodes for Long-Term Recordings in Deep Brain Centers." PLoS One **11**(5): e0155109.

Escamilla-Mackert T, Langhals NB, Kozai TD and Kipke DR (2009). "Insertion of a three dimensional silicon microelectrode assembly through a thick meningeal membrane." Conf Proc IEEE Eng Med Biol Soc 2009: 1616-1618.

Felix SH, Shah KG, Tolosa VM, Sheth HJ, Tooker AC, Delima TL, Jadhav SP, Frank LM and Pannu SS (2013). "Insertion of flexible neural probes using rigid stiffeners attached with biodissolvable adhesive." J Vis Exp (79): e50609.

Fontanini A and Katz DB (2005). "7 to 12 Hz activity in rat gustatory cortex reflects disengagement from a fluid self-administration task." J Neurophysiol **93**(5): 2832-2840.

Fu TM, Hong G, Zhou T, Schuhmann TG, Viveros RD and Lieber CM (2016). "Stable long-term chronic brain mapping at the single-neuron level." Nat Methods **13**(10): 875-882.

Godlove DC, Maier A, Woodman GF and Schall JD (2014). "Microcircuitry of agranular frontal cortex: testing the generality of the canonical cortical microcircuit." J Neurosci **34**(15): 5355-5369.

Guo ZV, Li N, Huber D, Ophir E, Gutnisky D, Ting JT, Feng G and Svoboda K (2014). "Flow of cortical activity underlying a tactile decision in mice." Neuron **81**(1): 179-194.

Hampson RE, Pons TP, Stanford TR and Deadwyler SA (2004). "Categorization in the monkey hippocampus: a possible mechanism for encoding information into memory." Proc Natl Acad Sci U S A **101**(9): 3184-3189.

Han X, Qian X, Bernstein JG, Zhou HH, Franzesi GT, Stern P, Bronson RT, Graybiel AM, Desimone R and Boyden ES (2009). "Millisecond-timescale optical control of neural dynamics in the nonhuman primate brain." Neuron **62**(2): 191-198.

Han M, Manoonkitiwongsa PS, Wang CX and McCreery DB (2012). "In vivo validation of custom-designed silicon-based microelectrode arrays for long-term neural recording and stimulation." IEEE Trans Biomed Eng **59**(2): 346-354.

Huang JY, Wang C and Dreher B (2017). "Silencing "Top-Down" Cortical Signals Affects Spike-Responses of Neurons in Cat's "Intermediate" Visual Cortex." Front Neural Circuits **11**: 27.

Jackson A and Fetz EE (2007). "Compact movable microwire array for long-term chronic unit recording in cerebral cortex of primates." J Neurophysiol **98**(5): 3109-3118.

Johnson MD, Franklin RK, Gibson MD, Brown RB and Kipke DR (2008). "Implantable microelectrode arrays for simultaneous electrophysiological and neurochemical recordings." J Neurosci Methods **174**(1): 62-70.

Kayser C, Wilson C, Safaai H, Sakata S and Panzeri S (2015). "Rhythmic auditory cortex activity at multiple timescales shapes stimulus-response gain and background firing." J Neurosci **35**(20): 7750-7762.

Kisley MA and Gerstein GL (1999). "Trial-to-trial variability and state-dependent modulation of auditory-evoked responses in cortex." J Neurosci **19**(23): 10451-10460.

Kondabolu K, Roberts EA, Bucklin M, McCarthy MM, Kopell N and Han X (2016). "Striatal cholinergic interneurons generate beta and gamma oscillations in the corticostriatal circuit and produce motor deficits." Proc Natl Acad Sci U S A **113**(22): E3159-3168.

Kozai TD, Du Z, Gugel ZV, Smith MA, Chase SM, Bodily LM, Caparosa EM, Friedlander RM and Cui XT (2015a). "Comprehensive chronic laminar single-unit, multi-unit, and local field potential recording performance with planar single shank electrode arrays." J Neurosci Methods **242**: 15-40.

Kozai TD, Catt K, Li X, Gugel ZV, Olafsson VT, Vazquez AL and Cui XT (2015b). "Mechanical failure modes of chronically implanted planar silicon-based neural probes for laminar recording." Biomaterials **37**: 25-39.

Kozai TD, Catt K, Du Z, Na K, Srivannavit O, Haque RU, Seymour J, Wise KD, Yoon E and Cui XT (2016). "Chronic In Vivo Evaluation of PEDOT/CNT for Stable Neural Recordings." IEEE Trans Biomed Eng **63**(1): 111-119.

Lasztóczi B and Klausberger T (2016). "Hippocampal Place Cells Couple to Three Different Gamma Oscillations during Place Field Traversal." Neuron **91**(1): 34-40.

Lee SE, Jun SB, Lee HJ, Kim J, Lee SW, Im C, Shin HC, Chang JW and Kim SJ (2012). "A Flexible Depth Probe Using Liquid Crystal Polymer." IEEE Trans Biomed Eng **59**(7): 2085-2094.

Lee HC, Gaire J, Roysam B and Otto KJ (2017). "Placing Sites on the Edge of Planar Silicon Microelectrodes Enhances Chronic Recording Functionality." IEEE Trans Biomed Eng doi: 10.1109/TBME.2017.2715811

Lee HC, Gaire J, McDowell SP and Otto KJ (2014) "The effect of site placement within silicon microelectrodes on the long-term electrophysiological recordings." Conf Proc IEEE Eng Med Biol Soc 2014:465-468.

Leiser SC, and Moxon KA (2006). "Relationship between physiological response type (RA and SA) and vibrissal receptive field of neurons within the rat trigeminal ganglion." J Neurophysiol **95**(5): 3129-3145.

Li X, Yamawaki N, Barrett JM, Kording KP and Shepherd GMG (2018). "Scaling of optogenetically evoked signaling in a higher-order corticocortical pathway in the anesthetized mouse." bioRxiv. doi: 10.1101/154914

Lim Y, Lagoy R, Shinn-Cunningham BG and Gardner TJ (2016). "Transformation of temporal sequences in the zebra finch auditory system" eLife **5**: e18205.

Maris E, Womelsdorf T, Desimone R and Fries P (2013). "Rhythmic neuronal synchronization in visual cortex entails spatial phase relation diversity that is modulated by stimulation and attention." Neuroimage **74**: 99-116.

McAlinden N, Gu E, Dawson MD, Sakata S and Mathieson K (2015). "Optogenetic activation of neocortical neurons in vivo with a sapphire-based micro-scale LED probe." Front Neural Circuits **9**: 25.

McGinty VB and Grace AA (2008). "Selective activation of medial prefrontal-to-accumbens projection neurons by amygdala stimulation and Pavlovian conditioned stimuli." Cereb Cortex **18**(8): 1961-1972.

Mechler F, Victor JD, Ohiorhenuan I, Schmid AM and Hu Q (2011). "Three-dimensional localization of neurons in cortical tetrode recordings." J Neurophysiol **106**(2): 828-848.

Mols K, Musa S, Nuttin B, Lagae L and Bonin V (2017). "In vivo characterization of the electrophysiological and astrocytic responses to a silicon neuroprobe implanted in the mouse neocortex." Sci Rep **7**(1): 15642.

Moxon KA, Kalkhoran NM, Markert M, Sambito MA, McKenzie JL and Webster JT (2004). "Nanostructured surface modification of ceramic-based microelectrodes to enhance biocompatibility for a direct brain-machine interface." IEEE Trans Biomed Eng **51**(6): 881-889.

Musall S, Haiss F, Weber B and von der Behrens W (2017). "Deviant Processing in the Primary Somatosensory Cortex." Cereb Cortex **27**(1): 863-876.

Neto JP, Lopes G, Frazao J, Nogueira J, Lacerda P, Baiao P, Aarts A, Andrei A, Musa S, Fortunato E, Barquinha P and Kampff AR (2016). "Validating silicon polytrodes with paired juxtacellular recordings: method and dataset." J Neurophysiol **116**(2): 892-903.

Nicolelis MA, Ghazanfar AA, Faggin BM, Votaw S and Oliveira LM (1997)."Reconstructing the engram: simultaneous, multisite, many single neuron recordings." Neuron **18**(4): 529-537.

Nicolelis MA, Dimitrov D, Carmena JM, Crist R, Lehew G, Kralik JD and Wise SP (2003). "Chronic, multisite, multielectrode recordings in macaque monkeys." Proc Natl Acad Sci U S A **100**(19): 11041-11046.

Oliveira-Maia AJ, Simon SA and Nicolelis MAL (2008). "Neural Ensemble Recordings from Central Gustatory-Reward Pathways in Awake and Behaving Animals." In: Nicolelis MAL, (editor), "Methods for Neural Ensemble Recordings." 2nd edition. Boca Raton (FL): CRC Press/Taylor & Francis, 2008. Chapter 10.

Okun M, Lak A, Carandini M and Harris KD (2016). "Long Term Recordings with Immobile Silicon Probes in the Mouse Cortex." PLoS One **11**(3): e0151180.

O'Shea DJ and Shenoy KV (2018). "ERAASR: an algorithm for removing electrical stimulation artifacts from multielectrode array recordings." J Neural Eng **15**(2): 026020.

Paralikar KJ and Clement RS (2008). "Collagenase-aided intracortical microelectrode array insertion: effects on insertion force and recording performance." IEEE Trans Biomed Eng **55**(9): 2258-2267.

Prasad A, Xue QS, Dieme R, Sankar V, Mayrand RC, Nishida T, Streit WJ and Sanchez JC (2014). "Abiotic-biotic characterization of Pt/Ir microelectrode arrays in chronic implants." Front Neuroeng **7**: 2.

Raducanu BC, Yazicioglu RF, Lopez CM, Ballini M, Putzeys J, Wang S, Andrei A, Rochus V, Welkenhuysen M, Helleputte NV, Musa S, Puers R, Kloosterman F, Hoof CV, Fiáth R, Ulbert I and Mitra S (2017). "Time Multiplexed Active Neural Probe with 1356 Parallel Recording Sites." Sensors (Basel) **17**(10): E2388.

Ramrath L, Vogt S, Jensen W, Hofmann UG and Schweikard A (2009) "Computer and robot-assisted stereotaxy for high-precision small animal brain exploration." Biomed Tech (Berl). **54**(1):8-13.

Rennaker RL, Ruyle AM, Street SE and Sloan AM (2005). "An economical multi-channel cortical electrode array for extended periods of recording during behavior." J Neurosci Methods **142**(1): 97-105.

Rohatgi P, Langhals NB, Kipke DR and Patil PG (2009). "In vivo performance of a microelectrode neural probe with integrated drug delivery." Neurosurg Focus **27**(1): E8.

Sakata S (2016). "State-dependent and cell type-specific temporal processing in auditory thalamocortical circuit." Sci Rep **6**: 18873.

Sawahata H, Yamagiwa S, Moriya A, Dong T, Oi H, Ando Y, Numano R, Ishida M, Koida K and Kawano T (2016). "Single 5 μm diameter needle electrode block modules for unit recordings in vivo." Sci Rep **6**: 35806.

Scharf R, Tsunematsu T, McAlinden N, Dawson MD, Sakata S and Mathieson K (2016). "Depth-specific optogenetic control in vivo with a scalable, high-density muLED neural probe." Sci Rep **6**: 28381.

Scherberger H, Fineman I, Musallam S, Dubowitz DJ, Bernheim KA, Pesaran B, Corneil BD, Gilliken B and Andersen RA (2003). "Magnetic resonance image-guided implantation of chronic recording electrodes in the macaque intraparietal sulcus." J Neurosci Methods **130**(1): 1-8.

Schoenfeld TJ, Kloth AD, Hsueh B, Runkle MB, Kane GA, Wang SS and Gould E (2014). "Gap junctions in the ventral hippocampal-medial prefrontal pathway are involved in anxiety regulation." J Neurosci **34**(47): 15679-15688.

Scott KM, Du J, Lester HA and Masmanidis SC (2012). "Variability of acute extracellular action potential measurements with multisite silicon probes." J Neurosci Methods **211**(1): 22-30.

Seidl K, Schwaerzle M, Ulbert I, Neves HP, Paul O and Ruther P (2012). "CMOS-Based High-Density Silicon Microprobe Arrays for Electronic Depth Control in Intracortical Neural Recording-Characterization and Application." J Microelectromech Syst **21**(6): 1426-1435.

Shen W, Karumbaiah L, Liu X, Saxena T, Chen S, Patkar R, Bellamkonda RV and Allen MG (2015). "Extracellular matrix-based intracortical microelectrodes: Toward a microfabricated neural interface based on natural materials" Microsystems & Nanoengineering **1**: 15010.

Shiramatsu TI, Takahashi K, Noda T, Kanzaki R, Nakahara H and Takahashi H (2016). "Microelectrode mapping of tonotopic, laminar, and field-specific organization of thalamo-cortical pathway in rat." Neuroscience **332**: 38-52.

Stolzberg D, Chrostowski M, Salvi RJ and Allman BL (2012). "Intracortical circuits amplify sound-evoked activity in primary auditory cortex following systemic injection of salicylate in the rat." J Neurophysiol **108**(1): 200-214.

Suyatin DB, Wallman L, Thelin J, Prinz CN, Jorntell H, Samuelson L, Montelius L and Schouenborg J (2013). "Nanowire-based electrode for acute in vivo neural recordings in the brain." PLoS One **8**(2): e56673.

Thimm A and Funke K (2015). "Multiple blocks of intermittent and continuous theta-burst stimulation applied via transcranial magnetic stimulation differently affect sensory responses in rat barrel cortex." J Physiol **593**(4): 967-985.

Venkatachalam S, Fee MS and Kleinfeld D (1999). "Ultra-miniature headstage with 6-channel drive and vacuum-assisted micro-wire implantation for chronic recording from the neocortex." J Neurosci Methods **90**(1): 37-46.

Wang J, Wagner F, Borton DA, Zhang J, Ozden I, Burwell RD, Nurmikko AV, van Wagenen R, Diester I and Deisseroth K (2012). "Integrated device for combined optical neuromodulation and electrical recording for chronic in vivo applications." J Neural Eng **9**(1): 016001.

Ward MP, Rajdev P, Ellison C and Irazoqui PP (2009). "Toward a comparison of microelectrodes for acute and chronic recordings." Brain Res **1282**: 183-200.

Wiebe SP and Staubli UV (1999). "Dynamic filtering of recognition memory codes in the hippocampus." J Neurosci **19**(23): 10562-10574.

Wiest M, Thomson E and Meloy J (2008). "Multielectrode Recordings in the Somatosensory System." In: Nicolelis MAL, (editor), "Methods for Neural Ensemble Recordings." 2nd edition. Boca Raton (FL): CRC Press/Taylor & Francis, 2008. Chapter 6.

Yamamoto J and Wilson MA (2008). "Large-scale chronically implantable precision motorized microdrive array for freely behaving animals." J Neurophysiol **100**(4): 2430-2440.

Yang H, Kwon SE, Severson KS and O'Connor DH (2016). "Origins of choice-related activity in mouse somatosensory cortex." Nat Neurosci **19**(1): 127-134.

Zeitler M, Fries P and Gielen S (2006). "Assessing neuronal coherence with single-unit, multi-unit, and local field potentials." Neural Comput **18**(9): 2256-2281.

Zhang S, Yen SC, Xiang Z, Liao LD, Kwong DL and Lee C (2015). "Development of Silicon Probe With Acute Study on In Vivo Neural Recording and Implantation Behavior Monitored by Integrated Si-Nanowire Strain Sensors." J Microelectromech Syst **24**(5): 1303-1313.

Zhang S, Song Y, Wang M, Xiao G, Gao F, Li Z, Tao G, Zhuang P, Yue F, Chan P and Cai X (2018). "Real-time simultaneous recording of electrophysiological activities and dopamine overflow in the deep brain nuclei of a non-human primate with Parkinson’s disease using nano-based microelectrode arrays." Microsystems & Nanoengineering **4**: 17070.

Zhao Z, Gong R, Huang H and Wang J (2016). "Design, Fabrication, Simulation and Characterization of a Novel Dual-Sided Microelectrode Array for Deep Brain Recording and Stimulation." Sensors (Basel) **16**(6): E880.

Supplementary Table S2 – Results of the single unit yield, peak-to-peak amplitude of single units and isolation distance of unit clusters after analyzing shortened (30-min-long) recordings. In the case of data obtained after the fastest (1 mm/s) insertion speed, the first 15 minutes were removed, which is the time period needed to insert the probe with the slowest (0.002 mm/s) insertion speed. In the case of recordings acquired after the slowest insertions, we removed the last 15 minutes to obtain recordings of equal lengths (30 minutes). After that, we performed spike sorting on the shortened data and calculated the single unit yield, the isolation distance and the peak-to-peak amplitudes the same way as we did for the original, 45-min-long recordings.

	**0.002 mm/s**	**1 mm/s**
**Total number of separated single units**	376	148
**Number of separated single units per penetration**	37.6 ± 13.9	16.4 ± 8.2
**Peak-to-peak amplitude (μV)**	197.5 ± 102.6	142.7 ± 64.7
**Isolation distance**	47.0 ± 54.8	27.1 ± 25.3

Supplementary Table S3 – Comparison of the properties of single units obtained from the whole electrode array of the 128-channel probe and units located in layer V. A single units was considered a layer V neuron if it had its largest amplitude spike waveform on a recording site located in layer V. The position of the recording sites relative to layer V was estimated by examining the coronal brain sections. The single unit yield in layer V was still inversely proportional to the insertion speed and still significantly different between the fastest and the slowest speed ($p = 0.049$; Kruskal-Wallis test). Furthermore, for the same speed, both the peak-to-peak amplitude of the spike waveforms and the first spike latency were similar between the two conditions. Average and standard deviation is presented.

**Properties**	**0.002 mm/s**	**0.02 mm/s**	**0.1 mm/s**	**1 mm/s**
Total number of separated single units	341	242	159	128
Number of layer V units	199	150	112	93
Number of separated single units per experiment	34.1 ± 12.2	24.2 ± 4.9	15.9 ± 7.9	14.2 ± 4.4
Number of layer V units per experiment	19.9 ± 8.2	15.0 ± 7.0	11.2 ± 8.0	10.3 ± 4.2
Peak-to-peak amplitude of all units (µV)	182.1 ± 99.4	142.1 ± 71.6	127.1 ± 59.6	137.3 ± 63.0
Peak-to-peak amplitude of layer V units (µV)	177.5 ± 96.6	146.2 ± 69.9	135.5 ± 66.4	139.3 ± 64.2
First spike latency of all units (s)	110.9 ± 246.0	209.5 ± 325.5	210.8 ± 329.9	294.4 ± 284.4
First spike latency of layer V units (s)	139.5 ± 290.7	185.3 ± 279.3	235.2 ± 358.3	290.7 ± 290.7

Supplementary Table S4 - Sequence of the insertion speeds used during each experiment carried out with the 128-channel silicon probe.

Animal	Left craniotomy		Right craniotomy	
	Speed of 1st insertion (mm/s)	Speed of 2nd insertion (mm/s)	Speed of 1st insertion (mm/s)	Speed of 2nd insertion (mm/s)
1	1	0.02	0.1	0.002
2	0.1	0.02	0.002	1
3	0.02	0.1	1	0.002
4	0.02	1	0.1	0.002
5	1	0.1	0.002	0.02
6	0.1	0.02	0.002	1
7	0.02	1	0.002	0.1
8	0.002	0.02	0.1	1
9	1	0.002	0.02	0.1
10	1	0.1	0.02	0.002

Supplementary Table S5 - Sequence of the insertion speeds used during the experiments with the 32-channel silicon probe.

Animal	Left craniotomy		Right craniotomy	
	Speed of 1st insertion (mm/s)	Speed of 2nd insertion (mm/s)	Speed of 1st insertion (mm/s)	Speed of 2nd insertion (mm/s)
1	1	0.002	0.002	1
2	0.002	1	1	0.002
3	1	0.002	0.002	1
4	0.002	0.002	1	1
5	1	1	0.002	0.002

Supplementary Table S6 – The number and ratio of single units recorded with the 128-channel probe which were excluded from the analysis.

	0.002 mm/s	0.02 mm/s	0.1 mm/s	1 mm/s	Sum/Ratio
**Number of single units included in the analysis**	341	242	159	128	∑ 870
**Number of units excluded by the amplitude criterion**	7	25	36	24	∑ 92
**Ratio of units excluded by the amplitude criterion (%)**	2,01	9,36	18,46	15,79	9,56%
**Units excluded by the violation rate criterion**	0	2	1	0	∑ 3
**Ratio of units excluded by the violation rate criterion (%)**	0	0,82	0,63	0	0,34%

Supplementary Table S7 – The number and ratio of single units recorded with the 32-channel probe which were excluded from the analysis.

	0.002 mm/s	1 mm/s	Sum/Ratio
**Number of single units included in the analysis**	220	157	∑ 377
**Number of units excluded by the amplitude criterion**	6	26	∑ 32
**Ratio of units excluded by the amplitude criterion (%)**	2,65	14,21	7,82%
**Units excluded by the violation rate criterion**	1	0	∑ 1
**Ratio of units excluded by the violation rate criterion (%)**	0,45	0	0,26%

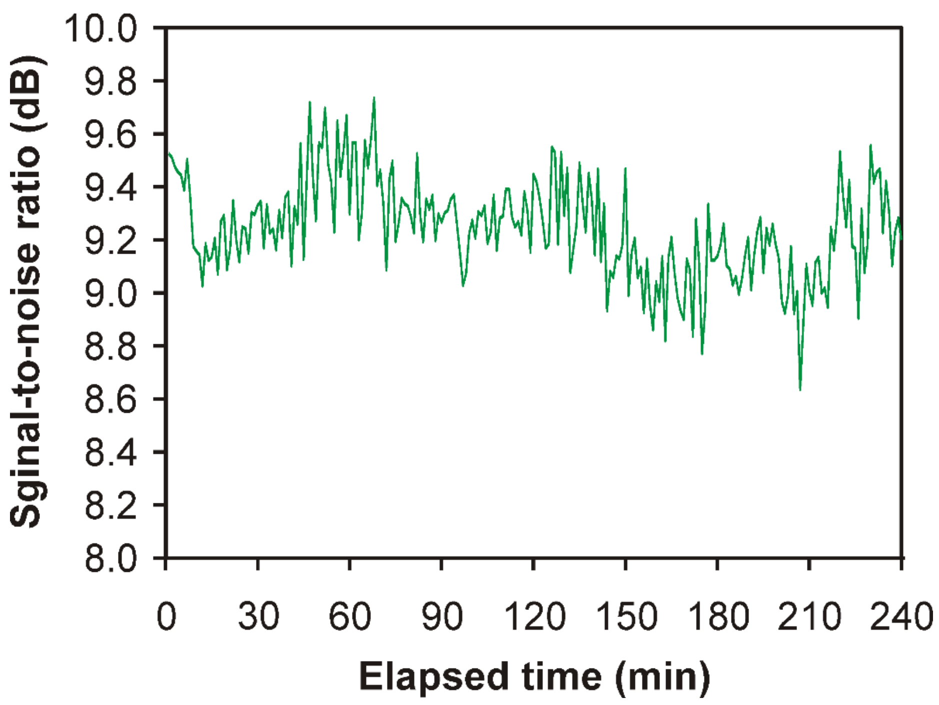

Supplementary Figure S1 – Change in the average SNR of the recorded spiking activity over time after inserting the 128-channel probe with slow (0.002 mm/s) speed for four hours (data of a single experiment). The SNR values were calculated from consecutive, 60-second-long segments of the recording, during the entire 240-minute-long recording period, then averaged across channels. The SNR stayed between 8.8 and 9.6 dB during the four-hour recording period.

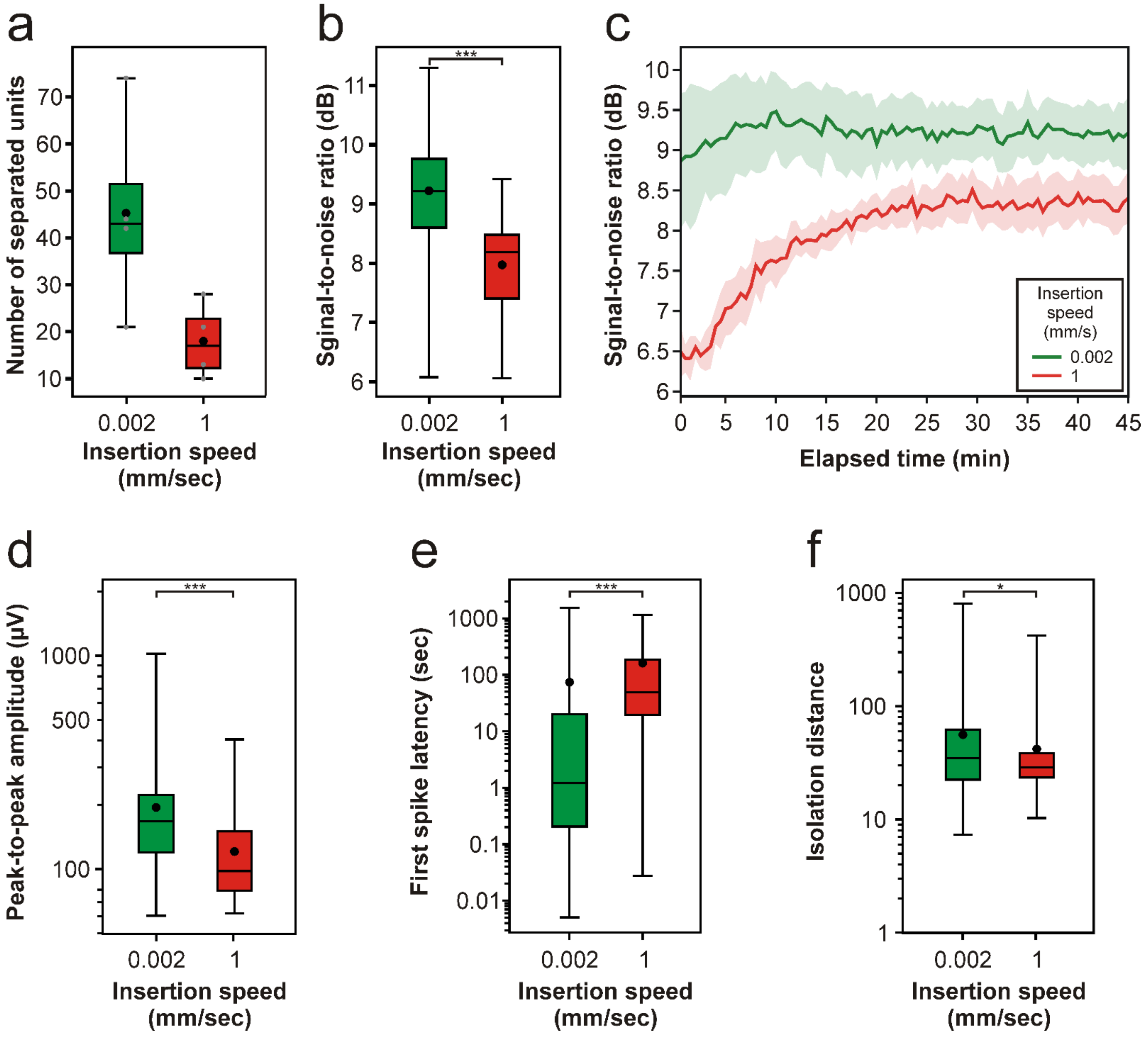

Supplementary Figure S2 – Properties of the single-unit activity recorded with the 128-channel silicon probe in experiments performed for histological investigation. (a) Box-and-whisker plot showing the distribution of the number of well-separated single unit clusters. (b) Box-and-whisker plot of the signal-to-noise ratio (SNR) values for each insertion speed. SNR values were calculated from consecutive, 30-second-long segments of the recordings, during the entire 45-minute-long recording period, then averaged across channels (number of computed SNR values after data cleansing for each speed: 0.002 mm/s, n = 358; 1 mm/s, n = 358). (c) Change in the average SNR of the recorded spiking activity over time for each insertion speed. Colored bands correspond to the standard error of mean. (d-f) Box-and-whisker plot showing the distribution of the peak-to-peak amplitude of spike waveforms (d), the distribution of the first spike latencies (e), and the distribution of the isolation distances (f) for each insertion speed (total number of well-separated neurons for each speed: 0.002 mm/s, n = 181; 1 mm/s, n = 72). On the box-and-whisker plots, the middle line indicates the median, while the boxes correspond to the 25th and 75th percentile. Whiskers mark the minimum and maximum values. The average is depicted with a black dot. Gray dots on panel (a) correspond to single unit yields obtained for individual penetrations. Data on panels (d-f) are plotted on a logarithmic scale. * $p < 0.05$; *** $p < 0.001$; Mann-Whitney U test. Number of units excluded from analysis based on the amplitude and violation rate criteria: 25 (0.002 mm/s) and 36 (1 mm/s).

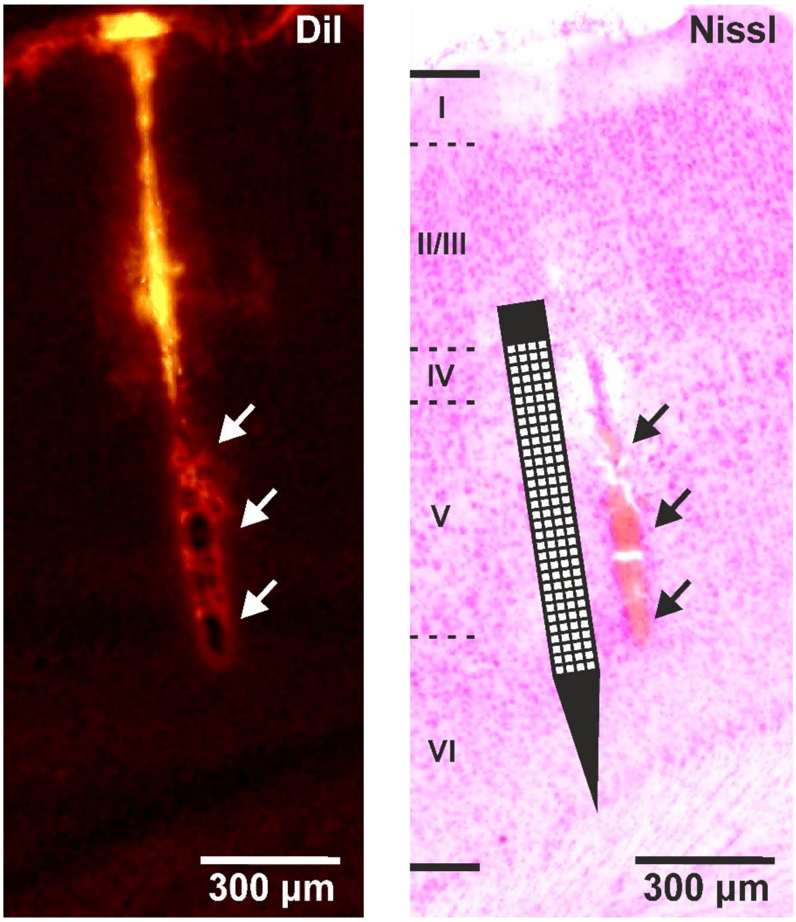

Supplementary Figure S3 – Coronal brain section showing the probe track after one of insertions carried out with the slowest (0.002 mm/s) speed (left side: track stained by DiI fluorescent dye; right side: brain section after Nissl-staining). The acquired electrophysiological recording was the only one among the recordings obtained after the slowest insertions where only a very low number of single units (n = 4) were detected after probe insertion (average single unit yield after the slowest insertions: 34.1 units). On this brain section, signs of blood were identified next to the probe track (indicated by arrows). The traces of blood were located mostly in layer V, from where most of the electrodes of the probe recorded the neuronal activity (see the probe schematic next to the track). Dashed lines mark cortical layer boundaries.
